# Multivariable reference centiles for maximum grip strength in childhood to young adults

**DOI:** 10.1038/s41430-023-01395-4

**Published:** 2023-12-29

**Authors:** Ibrahim Duran, Kim Ramona Wloka, Kyriakos Martakis, Karoline Spiess, Ute Alexy, Eckhard Schoenau

**Affiliations:** 1 University of Cologne, Medical Faculty and University Hospital, Center of Prevention and Rehabilitation, UniReha, Cologne Germany; 2 University of Cologne, Medical Faculty and University Hospital, Department of Pediatrics, Cologne, Germany; 3 Department of Pediatric Neurology, University Children’s Hospital and Medical Faculty, Justus Liebig University of Giessen, Giessen, Germany; 4 Institute of Nutritional and Food Sciences, Nutritional Epidemiology, DONALD Study, University of Bonn, Dortmund, Germany

**Keywords:** Biomarkers, Paediatrics, Skeletal muscle, Risk factors, Physical examination

## Abstract

**Objectives:**

Maximum grip strength (mGS) is a useful predictor of health-related outcomes in children and adults. The aim of the study was to generate sex- and age-adjusted reference centiles for mGS for children, adolescents and young adults, while adjusting for body height and body mass index (BMI).

**Methods:**

A retrospective analysis of longitudinal data from children and young adults participating in the DOrtmund Nutritional and Anthropometric Longitudinally Designed (DONALD) study (single center, open cohort study) from 2004 to 2022 was conducted. To generate sex-, age-, height- and BMI-adjusted reference centiles, a new algorithm combining multiple linear regression and the LMS method was conducted.

**Results:**

Overall, 3325 measurements of mGS of 465 females and 511 males were eligible. The mean age at measurement of females was 12.6 ± 3.9 years, mean age of males was 12.4 ± 4.7 years. The median of number of repeated measurements per individual was 3 (range 1–8). The mGS was significantly (*p* < 0.001) correlated to body height and BMI (*r* = 0.303–0.432). Additional reference centiles for the change of z-scores of mGS were generated for children and young adults from 8 to 20 years.

**Conclusions:**

We proposed to evaluate mGS in children, adolescents and young adults with the presented reference centiles adjusted to sex, age, height and BMI. The method presented may also be applicable to other biological variables that depend more than just on sex and age. For the first time, also reference centiles to assess the change of mGS in repeated measurements were presented.

## Introduction

Muscular strength has been shown to be a useful predictor of health-related outcomes in children and adults [1–3]. Grip strength (GS) has been used in many studies to operationalize muscular strength [4] because it is easy to measure and correlated with overall muscle strength [5, 6]. In healthy adolescents, GS was correlated with bone mineral density and content [1, 3]. One standard deviation (SD) increase in GS during childhood and young adulthood (9–36 years) was inversely associated with the risk of prediabetes or type 2 diabetes in mid-adulthood (38–49 years) [2]. In addition, low GS in adolescents was associated with higher cardiometabolic risk and might be useful to identify who would benefit from lifestyle interventions [7].

To accurately evaluate the GS in childhood and adolescents, sex- and age-adjusted reference centiles are needed, because GS showed a different development in girls and boys [8–10]. The GS also depended on the body height and mass or body mass index (BMI) [11]. There were some attempts to develop normative data for children considering sex, age, body height and mass. Ploegmakers et al. [8] proposed a multiple linear regression line using a multilevel analysis that included sex, age, height and body mass in the formula. Kocher et al. [12] proposed a correction formula for the GS using an allometric scaling exponents for height and body mass, which was age- and sex-adjusted.

When generating reference centiles it is important that the z-scores for the reference population is not only standard normal distributed (mean = 0, standard deviation = 1.0) in total, but also conditional on the values of the explanatory variable (in this case age, height and body mass) [13]. Only if this statistical condition is met, is it ensured that 3% of the reference population have values below the 3^rd^ centile. Thus, in clinical use children and adolescents can be classified as conspicuous low because their GS in relation to all relevant influencing factors is lower than 3% of the reference population [14]. To our knowledge, until today there are no reference centiles for GS for children and young adults fulfilling these conditions.

Some authors supposed that a decline in GS is stronger predictor for some health-related outcomes than a single observation of low GS. For example in ageing women, “becoming weaker” was better predictor of falling, physical disability and frailty than “being weak” [15].

Therefore, it seems important that when GS is measured longitudinally, the change from the last measurement is also evaluated. The question to be answered is whether the difference in a z-score is clinically relevant, or whether it is rather an irrelevant fluctuation. To our knowledge, there are currently no normative data for this question.

Unfortunately, there is currently no comparable study in children and adolescents that examines whether a significant declines in GS during childhood/adolescence can translate into negative effects on health.

Hence, the aims of the study were to generate sex-, age-, height-, and BMI-adjusted reference centiles for maximum GS (mGS) and to establish reference centiles to evaluate differences in z-scores for repeated mGS measurements in children, adolescents and young adults. We also aimed to evaluate the level of association of the mGS with anthropometric variables (height, BMI).

## Methods

### Study design and study population

The DONALD study is a German open cohort study starting in 1985 with 1800 participants to the end of 2021 (age: three month to adulthood) [16]. Every year a small group of infants from the metropolitan Dortmund are recruited. These children participate in observational study visits throughout infancy, childhood and adolescence until adulthood. Within the framework of the DONALD study, detailed data on nutritional intake, growth, development, metabolism, and health status are collected at regular, usually annual intervals [17]. These data can be used to examine the diet-health or growth-health relationships, but also to create reference data from healthy children and adolescents [17]. Since 2012, the study is affiliated with the Department of Nutritional Epidemiology of the Institute for Nutrition and Food Sciences (IEL) of the Rheinische Friedrich-Wilhelms University of Bonn. The study is registered in the German Clinical Trial Register (DRKS00029092). Informed consent from the legal representatives of the participants were obtained.

A total of 3490 GS measurements were available from assessments. For 160 measurements, there were less than three individual measurements during a visit and for a further 5 assessments no body height data were available. These were excluded from the study. Hence, a total of 3,325 measurements were included in the analysis of which the highest value of the three repeated measurements was used for further analyses (mGS).

### GS and anthropometric measurements

Since 2004, mGS was measured on the non-dominant hand using a hydraulic dynamometer (Jamar ® Jackson, MI, 49203 USA). Children from age of six years were examined every two years, and young adults (≥18 years) on every 5-year visit.

At each visit, three repeated measures of the mGS were performed. At each visit, three repeated measures of the mGS were performed and short-term pause between measurements was considered. The subjects sat in an upright position with their feet flat on the floor and neutral position of the shoulder, elbow flexion 90°, forearm neutral position, wrist extension 0° to 30°. The subjects were asked to squeeze the dynamometer as hard as possible so that the mGS could be read off.

Body height was recorded in upright standing position with an approximation of 0.1 cm using a digital telescopic wall-mounted stadiometer (Harpenden, Rappenswil, Switzerland). Body mass was measured with an electronic scale (Model 753 E; Seca, Hamburg, Germany) at approximation of 0.1 kg [17]. Body mass index (BMI) was defined as $\frac{{weight}({kg})}{{{height}(m)}^{2}}$. Z-scores of height and BMI were calculated using the reference centiles of Neuhauser et al. [18].

The study sample of this analysis consisted of all data collected between 2004 to 2022.

### Statistical analysis

The descriptions of the statistical analysis have been moved to the supplements because they are complex and detailed descriptions were too long for the main part of the article.

## Results

### Study population

The data were derived from 976 children and young adults until the age of 24 years from 726 different families (mean age for females 12.6 ± 3.9 years, *n* = 465 and for males 12.4 ± 4.7 years, *n* = 511).

The range for z-scores for height in female was from −3.2 to 3.6, and for male from −3.4 to 4.2. The range for z-scores for BMI in female was from −3.3 to 2.7 and in male from −3.9 to 3.2. Further details of the study population were given in Table 1.

**Table 1:** Study population.

	Female	Male
Participants, *n*	465	511
GS measurements, *n*	1584	1741
Measurements per individual, median (range)	3 (1–8)	3 (1–8)
Age, years	12.6 (3.9)	12.4 (4.7)
Height, cm	150.9 (19.6)	155.1 (24.0)
Height, Z-Score	0.4 (0.96)	0.41 (1.03)
BMI, kg/m^2^	19.2 (3.7)	19.3 (4.1)
BMI, Z-Score	−0.05 (0.89)	−0.02 (0.96)

All values are mean (SD) unless otherwise stated.

*BMI* body mass index, *GS* grip strength.

### Sex- and age-adjusted reference centiles for mGS

**Fig. 1:**
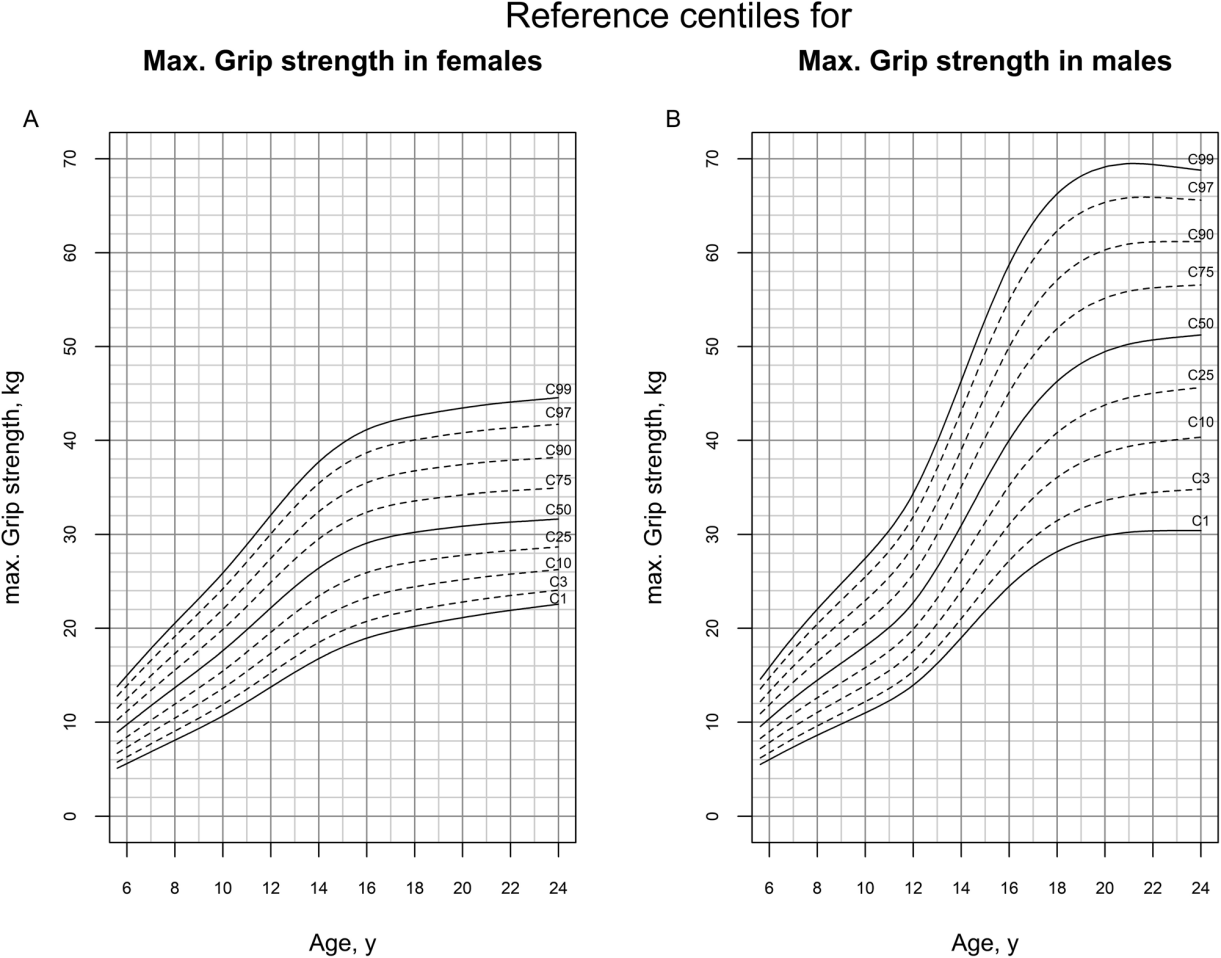
Reference centiles for mGS. Age-adjusted reference centiles for mGS were presented for females (**A**) and males (**B**).

Figure 1 depicts the sex- and age-adjusted reference centiles for mGS. The tabulated reference centiles are given in Supplementary Tables 1 and 2. Z-scores for height were significantly positive correlated with z-scores for mGS (adjusted for sex and age, *p* < 0.001, for female *r* = 0.432 (95% CI 0.391–0.471), for male *r* = 0.399 (95% CI 0.359–0.438)).

**Fig. 2:**
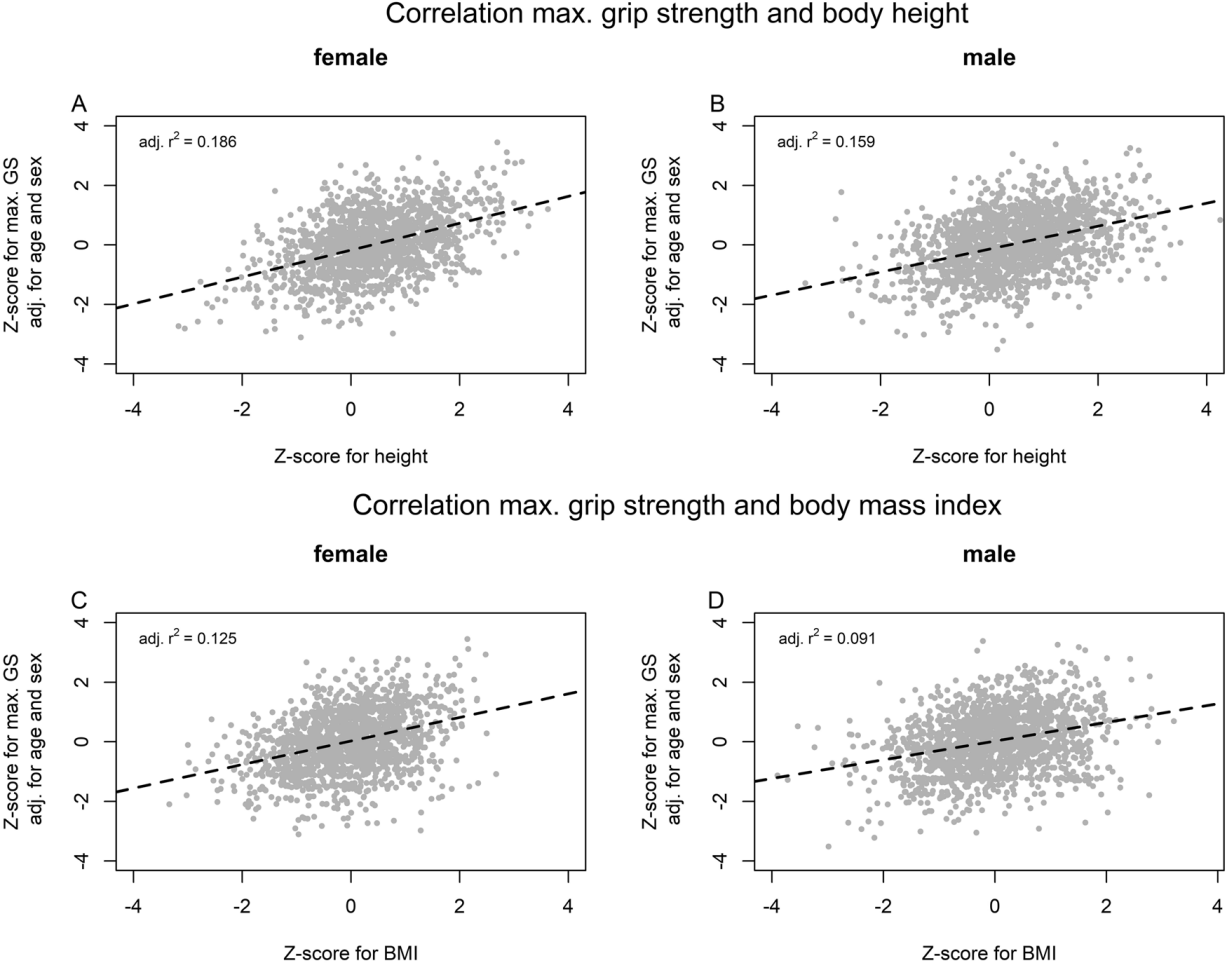
The correlation between mGS and height respectively BMI. The correlation between z-scores for mGS (adj. for sex and age) and height for females (**A**) and males (**B**) were presented. The dashed line represented the linear regression line. The correlation between z-scores for mGS and body mass index (BMI) for females (**C**) and males (**D**) were presented. The dashed line represented the linear regression line.

Z-scores for BMI were significantly positive correlated with z-scores for mGS (*p* < 0.001, for female *r* = 0.354 (95% CI 0.310–0.396), for male *r* = 0.303 (95% CI 0.260–0.345)). The scatterplots between z-scores for mGS and for height and BMI are given in Fig. 2. Since z-scores for height and BMI were correlated only slightly in both sexes (for female *r* = 0.205 (95% CI 0.157–0.252), for male *r* = 0.108 (95% CI 0.062–0.154)), we dispensed with the additional calculation of partial correlation coefficients.

### Sex-, age-, height- and BMI-adjusted reference centiles for mGS

**Fig. 3:**
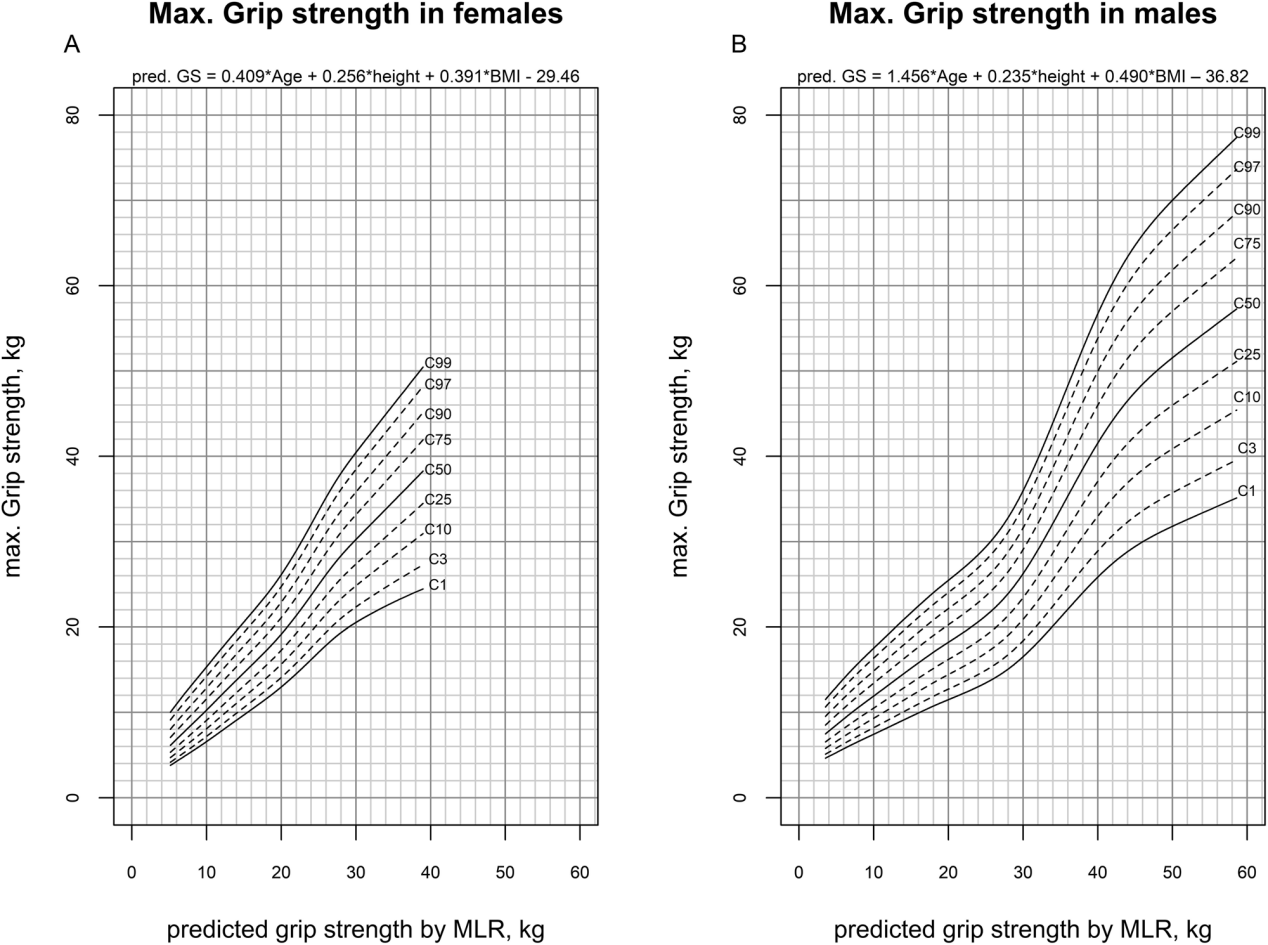
Multivariable reference centiles for mGS. Age-, height- and BMI-adjusted reference centiles for mGS were presented for females (**A**) and males (**B**). First, the predicted GS is determined using the multiple linear regression formula presented in the figure based on age, height and BMI. Then the predicted GS and the measured mGS are entered in the corresponding reference centile.

The multiple linear regression model (MLR) turned out to be an unbiased prediction of the mGS (pGS) since the mean of the Bland-Altman plot was near zero (Supplementary Fig. 1). Figure 3 depicts pGS-adjusted reference centiles for mGS. First, the predicted GS is determined using the multiple linear regression formula presented in the Fig. 3 based on age, height and BMI. Then the pGS and the measured mGS were entered in the corresponding reference centile. The tabulated reference centiles were given in Supplementary Tables 3 and 4.

The Q-Permutation test results are given in Supplementary Table 5. The test results indicated only a marginal significant deviation from the standard normality for BMI as explanatory variable in males (*p* = 0.043 for Q2-statistics which assess the standard deviation of the distribution). Visual analysis of the worm plots showed no relevant deviation from a standard normal distribution.

Only in females, z-scores for BMI were significantly (*p* < 0.001) slightly-positive correlated with z-scores for mGS (adj. to sex, age, height and BMI; *r* = 0.095 (95% CI 0.046–0.143)).

**Fig. 4:**
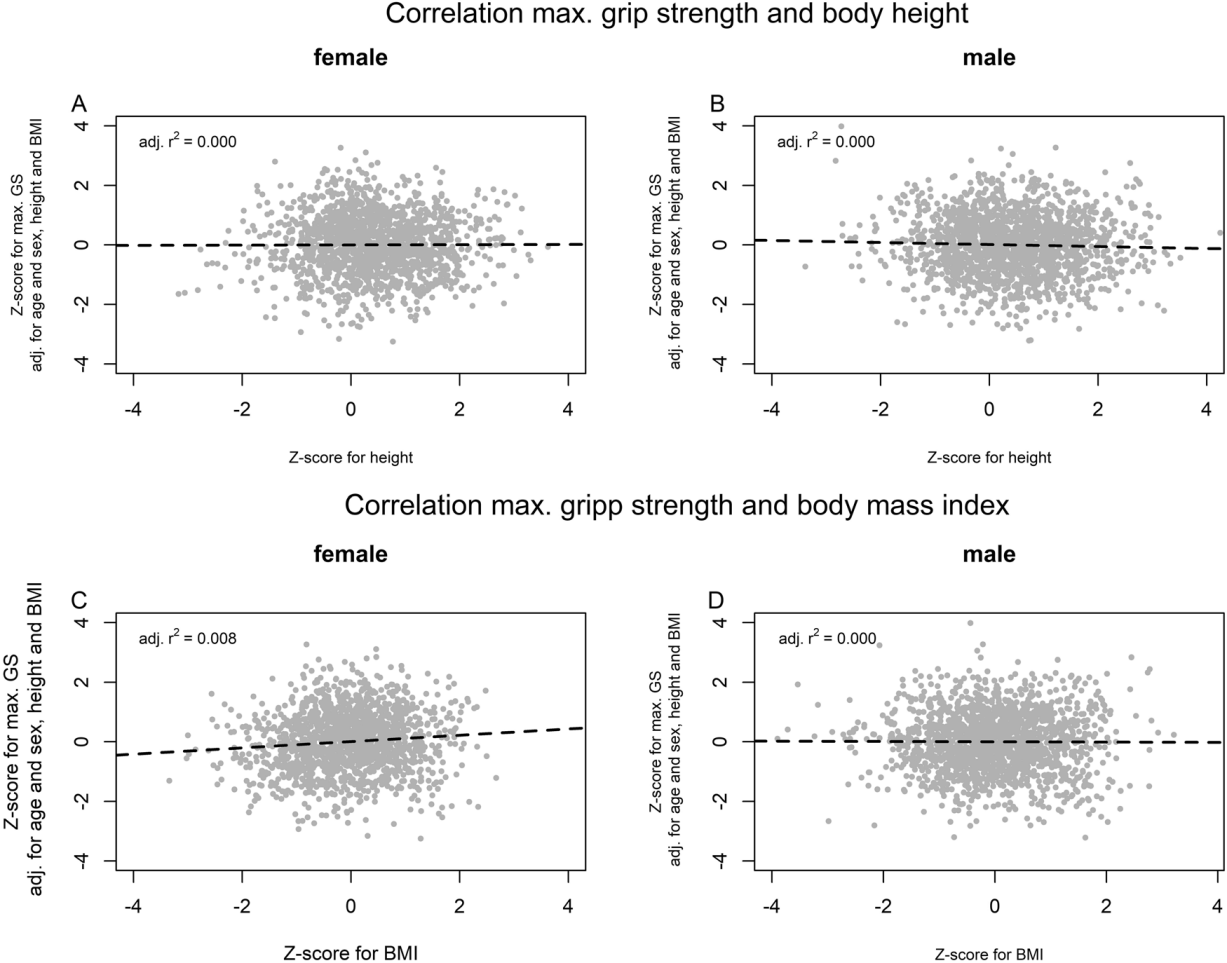
The correlation between mGS and height respectively BMI. The correlation between z-scores for mGS (adj. for sex, age, height and BMI) and height for females (**A**) and males (**B**) were presented. The dashed line represented the linear regression line. The correlation between z-scores for mGS and body mass index (BMI) for females (**C**) and males (**D**) were presented. The dashed line represented the linear regression line.

In both sexes, there were no significant correlation between z-scores for height (female: *p* = 0.893, male: *p* = 0.148) respectively BMI for male (*p* = 0.842) and z-scores for mGS (adj. for sex, age, height and BMI). Figure 4 depicts the scatterplots between z-scores of mGS and of height and BMI. Supplementary Figs. 2 and 3 depict the projections of the reference centiles of mGS with three explanatory variables on one with only age as explanatory variables.

### Reference centiles for differences in z-scores of repeated mGS measurements

The reference centiles for the annualized difference of z-scores of mGS are given in Supplementary Fig. 4. The tabulated reference centiles are given in Supplementary Tables 6–9. Since there was too little data after the age of 20 years, the reference centiles were only presented up to the age of 20 years.

### Clinical application of the sex-, age-, height- and BMI-adjusted z-scores of mGS

The results of the logistic regression are depicted in the Supplementary Fig. 5. An error rate of 10% was given with regard to the false negative classification (for low mGS) if the sum of the z-scores for height and BMI was below approximately −2.0 SD. With regard to the false negative classification, the 10% error rate was achieved from more than approximately 4.0 SD.

## Discussion

### Max. grip strength assessed by sex- and age-adjusted reference centiles

In this study, we present sex- and age-adjusted reference centiles for mGS in children and young adults. To our knowledge, these are the first pediatric reference centiles based on longitudinal data. Our results show, comparable with previous research [8, 9, 12] that GS increases in children and adolescents until their mid-twenties. In addition, girls reach a plateau earlier than boys and on average, boys have a higher mGS than girls after puberty (Fig. 1, 50th centile for males >50th centile for females).

Dodds et al. [9] combined data from 12 British studies to produce normative data for GS from 5 to 90 years (device: mostly Jamar, mostly in sitting, max. value of both hands). These data were slightly lower than the sex- and age-adjusted reference centiles calculated here (Supplementary Fig. 6A, B). One potential reason could be the poor temporal resolution with normative data at 5-year intervals (5–10–15–20 years). Our results are in accordance with the results of Ploemakers et al., who calculate normative data (mean and standard deviation) for GS in children from 4 to 14 years in Netherlands (Jamar, each hand separately, sitting, mean or max. was not stated, Supplementary Fig. 6C, D) [8]. The normative data of Kocher et al. in children (8–18 years in the USA) were also comparable to our results despite different measurement standards (Takei Digital GS dynamometer, standing position, both hands, mean, Supplementary Fig. 6E, F) [12].

When reporting motoric assessment results, it is important to describe the measurement standards. Important measurement standards for assessing GS are the used device (here: hydraulic dynamometer, Jamar), which hand is measured (dominant, non-dominant or both; here: non-dominant), which value is used (mean of all measurement or maximum; here: maximum) and the body position (standing or sitting; here: sitting) [9]. There are inconclusive data whether the measurement standard have an clinical relevant impact of the measurements results [9, 19, 20]. However, the majority of authors agree that if one wants to use a specific reference centile in clinical practice, the GS measurements should be made under the same measurement standards [21].

Since longitudinal data on mGS were available in this study, it was also possible (to our knowledge for the first time) to create reference centiles on the change in z-scores from the mGS in repeated measurements in children and young adults (Supplementary Fig. 4). This means that with the help of the presented reference centiles it is not only possible to assess a single measured mGS, but also the change in mGS from one measurement to next. There are some evidence in adults that in addition to a low value, a decrease in the mGS was also associated with negative health-related outcomes [15, 22]. Centile-crossing development (e.g. of the head circumference) is also a red flag in pediatric preventive examinations [23]. However, in clinical care it is often not clear how large a centile drop or z-score decrease must be in order to be considered conspicuous, since slight centile fluctuations are not uncommon. Centiles and z-scores can be converted into one another, but z-scores are more suitable for mathematical calculations. Using the presented reference centiles for the change in the z-scores for mGS (Fig. 4A, C, Supplementary Tables 6 and 7), it is possible to precisely determine how often such a change occurred in the reference population.

In Fig. 4A, C, it can be seen that the variability of the z-scores (for mGS) for both sexes decrease with increasing age. The cause could be that reasons for motor variability, such as growth spurts, decrease with age. Interestingly, the downward trend stagnated during puberty in females and in males there was even an increase, only to decrease again after puberty.

### Max. grip strength assessed by sex-, age-, height- and BMI-adjusted reference centiles

Our results confirm the positive correlation of mGS with height and BMI as described by other authors (Fig. 2) [8, 11, 12]. If this relationship is not taken into account, a low mGS, which is explained by a short height and/or a low BMI, could be incorrectly evaluated as conspicuous low (false positive). On the other hand, a mGS value that is too low in relation to age, height and BMI could be incorrectly assumed to be inconspicuous in a subject with a large height and/or high BMI (false negative).

For this purpose, we developed a method that is easy to use in clinical practice to assess the mGS adjusted for sex, age, height and BMI. First, the predicted GS (pGS) is determined using the MLR-formula presented here based on age, height and BMI (Fig. 3). Then the pGS and the measured mGS are entered in the corresponding reference centile (Fig. 3). Using the tabulated reference centiles in Supplementary Tables 3 and 4, exact z-scores can also be determined. The sex-, age-, height- and BMI-adjusted z-scores showed no relevant correlation with height or BMI anymore (Fig. 4). In addition, the z-scores were conditional normal distributed to all continuous explanatory variables (age, height and BMI), which is a necessary characteristic for reference centiles (Supplementary Table 5) [14].

Kocher et al. [12] created normative data for GS and proposed an allometric scaling of the measured GS. Indeed the allometric scaled GS was no longer correlated with height and body mass. The disadvantage of this work was that neither the reference centile was calculated using the LMS method (the most common method for generating smoothed reference percentiles), nor was the conditional normality of all explanatory variables controlled.

In order to visualize the presented multivariable reference centiles more clearly, averaged “projections” were generated for fixed ranges for height and BMI and compared with the classic, only sex- and age-adjusted, reference centiles (details see Methods). These projections are to be understood in a similar way to, for example, a projection of a three-dimensional cube onto a two-dimensional surface from a certain viewing direction. The illustrations in Supplementary Figs. 2 and 3 clearly show that the deviation (of the multivariable reference centiles) from the only sex- and age-adjusted reference centiles increased with increasing absolute values of the z-scores for height and BMI (as expected). It was observed that opposite deviations in the z-score for height and BMI (e.g. large height but low BMI) partially compensated each other in such a way that the sex-, age-, height- and BMI-adjusted reference centiles were similar to the only sex- and age-adjusted (see the diagonal sub-figures from left upper to right lower in Supplementary Figs. 2 and 3). On the other hand, we found that the deviations are greatest when z-scores for height and BMI were aligned (e.g. tall and heavy, or light and small, see sub-figures first row and last column or last row and first column, Supplementary Figs. 2 and 3).

Since the use of the multivariable reference centile is somewhat more complex, the question arose as to which patients it should definitely be used for and for which the sex- and age-adjusted one is sufficient. To do this, we analyzed the error rate of the only sex- and age-adjusted reference centiles in detecting low. max GS. As an example, we considered an error rate of 10% to be clinically relevant and sufficient to justify the additional effort of the multivariable reference centiles (other limits for error rate can also be calculated using the Supplementary Fig. 5). The sum of the z-scores for height and BMI seemed to be suitable as a decision criterion. Our analyzes supported the recommendation to apply the multivariable reference centiles if the total of the sum of the height and BMI z-scores is below −2.0 or above 4.0 (see Supplementary Fig. 5). In addition, with Supplementary Fig. 4B, D and Supplementary Tables 8 and 9, it is possible to evaluate the change in z-scores for mGS adjusted for sex-, age-, height- and BMI.

### Method of the multivariable reference centiles

There is a need for reference centiles with more than just one explanatory variable in pediatrics since many clinically relevant biological variables such as e.g. muscle mass and bone mass are not only dependent on sex and age, but also on height and BMI [24–26]. In literature, there are very different proposed approaches to handle this situation. Some authors, as described in introduction, suggest different adjustments of the raw measurement value [8, 12], others described an additional adjustment of the sex- and age-adjusted z-scores to overcome the association to height and BMI [26].

The developers of gamlss presented a method with two explanatory variables [27]. Here, however, the graphical representation consisted of special contour plots and, for reasons of practicability, only for a few selected centiles. A method with three explanatory variables was not described.

To our knowledge, the presented method for creating multivariable reference centiles for three explanatory variables has not been described before. The method presented is comparatively easy to use and contains a graphic representation as the clinicians are already familiar. In addition, in our study population and at the evaluation of mGS, this methodology succeeded to produce z-scores for mGS which was standard normal distributed conditional on all explanatory variables.

Only further studies can show whether there are comparable results in other populations and biological variables. Unfortunately, we cannot provide a formal mathematical proof under which condition this method leads regularly to the desired results.

### Limitations

Our study cohort presents a convenience sample of boys and girls from the metropolitan area of Dortmund, which may not represent the totality of German children. Only the non-dominant hand was assessed, while other factors that may influence GS (e.g., hand size or forearm girth; sports training; and nutritional status) were not evaluated [28].

### Conclusions

We propose the evaluation of mGS in children, adolescents and young adults with the presented reference centiles adjusted to sex, age, height and BMI, especially if the total of the sum of the height and BMI z-scores is below −2.0 or above 4.0. The method presented may be applicable to other biological variables that depend more than just on sex and age. For the first time, we generated reference centiles to assess the change of mGS in repeated measurements.

### Supplementary information

Supplement

Supplement Figure legends

Supplement Fig. 1

Supple. Fig. 2

Supple. Fig. 3

Supple. Fig. 4

Supple. Fig. 5

Supple. Fig. 6

## Data Availability

Data is available upon request to alexy@uni-bonn.de.
