# Supplementary material for: Multivariable reference centiles for maximum grip strength in childhood to young adults
Source: Eur J Clin Nutr. 2023 Dec 29;78(6):494–500. doi: 10.1038/s41430-023-01395-4 (PMC11182743; doi:10.1038/s41430-023-01395-4)
Supplement: Supplementary file 1 — Supplement [file 41430_2023_1395_MOESM1_ESM.docx]

# Supplement

## Statistical analysis

The descriptions of the statistical analysis have been moved to the supplements because they are complex and detailed descriptions were too long for the main part of the article.

### Sex- and age-adjusted reference centiles for mGS

Sex- and age-adjusted reference centiles were generated using the lamda-mu-sigma (LMS) method of Cole and Green [1]. The concrete implementation was done with the package *gamlss* [2] (version 5.4-12) for R (version 4.2.3) by using the *Box-cox Cole and Green* distribution (BCCG). The LMS method ensures that the median, standard deviation and skewness of the calculated z-scores for the reference population correspond to a standard normal distribution and is widely used to generate reference centiles. The goodness of fit of the model is assessed using suitable statistical tests (Q-test [3] and Worm plot [4]) and the model is improved by adjusting the degrees of freedom for the three parameters mentioned. For more details, please see the cited literature.

Although the LMS method (by *gamlss*) was developed for cross-sectional data, it is also used for longitudinal data when the number of repeat measurements is relatively small compared to the number of subjects [5]. An example of this are the WHO child growth standards (2006) [5].

For the evaluation of the association of mGS and anthropometric variables, scatterplots of the z-scores for mGS and z-scores for height and BMI were shown and Pearson correlation coefficients were calculated.

### Sex-, age-, height- and BMI-adjusted reference centiles for mGS

Unfortunately, *gamlss* cannot be simply used for the generating of reference centiles with more than one explanatory variables. Therefore, we want to developed a method for generating a reference centile for three explanatory variables (age, height and BMI) which fulfill following conditions:

1. easy to apply in clinical situation like a typical reference centile
2. z-scores of the reference population should be conditional normal to all explanatory variables and
3. z-scores and centiles should be calculated simply for future individuals.

The proposed algorithm is as follows

1. Using multiple linear regression (MLR) we developed a model to predict the grip strength (predicted grip strength, pGS) as a function of age, height and BMI for both sexes separately. With the help of the MLR, the three explanatory variables should be combined into one. The assumptions for the multiple linear regression were analyzed with QQ-plots (normal distribution of the residuals), “residuals-vs-fitted value” plots (homoscedasticity of the residuals), “residuals-vs-leverage” plots (outlier analysis) and the variance inflation factor (multicollinearity).
2. We generated a reference centile for mGS adjusted for pGS as described in the section before.

For the evaluation of the goodness of fit of this combined reference centile, the generated z-scores for mGS were tested for standard normal distribution conditional on separately age, height and BMI in both sexes separately using Worm plots[4] and the Q-Permutation test[6]. The Q-Permutation test is a variation of the Q-test[3], which only calculates Q-statistics and p-values for residuals of a gamlss-object. The Q-Permutation test evaluate the normality of any model residuals within a range of an independent variable and calculate an approximated p-value using a permutation approach. For more details on the Q-Permutation test see cited literature.

Again, scatterplots and Pearson correlation coefficients were used to evaluate the association of z-scores of mGS and height resp. BMI (s. section before).

To compare the reference centiles with three explanatory variables with the one with only age as explanatory variable, following approach was performed:

1. For a specified range of length z-scores and BMI z-scores (e.g. -0.5 to 0.5, corresponds to C30 and C70), the corresponding heights in cm and BMI value at C50 were calculated at a distance of 0.1 z-scores for height and BMI. These calculations were performed for ages from 6 to 24 in 0.5 year steps (sexes separately). For example, for the age “6 years”, there would be 11 steps for z-scores height and 11 steps for BMI from -0.5 to 0.5. This means 121 data points with (age, height, BMI).
2. The pGS was determined for each data points consisting of age, height and BMI by the MLR model.
3. With the determined pGS, the values for mGS at C1, C50 and C99 were determined using the pGS-adjusted reference centiles for mGS.
4. This led to a number of mGS (for C1, C50 and C99) values being calculated for each age step over the height and BMI range under consideration. The mean value of the determined mGSs was then assigned to the age step.

This creates a “projection” of the reference percentile with 3 explanatory variables onto one (age-adjusted). This should then be overlaid with the reference percentile with only age as an explanatory variable.

### Reference centiles for differences in z-scores of repeated mGS measurements

Only those participants who had more than one measurement of GS were considered for this analysis. In these participants, the difference in z-scores from consecutive measurements was determined. That means, a child with e.g. 3 measurements had 2 z-score differences.

The z-score difference usually referred to different time intervals, so that all were annualized to one year and assigned to the mean of the both ages of measurements. Then the reference centiles for z-score differences adjusted to the age were calculated separately for both sexes. Since the z-score differences could be smaller than zero the power exponential distribution was used (PE, three parameter distribution on (-∞, ∞), BCCG is not suitable for that purpose).

### Clinical application of the sex-, age-, height- and BMI-adjusted z-scores of mGS

As a rule, a maximum grip strength that is too high will not have any clinical consequences. Therefore, the finding of a too low mGS is clinically relevant. There are no generally accepted cutoffs from which a finding regarding a reference centile is defined as *conspicuous* *low*. Common cutoffs are the 10^th^, 5^th^ or 3^rd^ centiles. In this study, following definitions were made

- A *low mGS* was defined as a value lower than the 3^rd^ centile (equal to z-score < -1.88)
- An inconspicuous finding was defined as a value higher or equal than the 3^rd^ centile (equal to z-score ≥ -1.88)
- The gold standard for evaluating the mGS was defined as the centile (or z-score) according the reference centiles adjusted for sex, age, height and BMI.
- A false positive classification of low mGS was defined as a low mGS according the reference centiles adjusted to sex and age, and an inconspicuous mGS according the reference centiles adjusted to sex, age, height and BMI.
- A false negative classification of low mGS was defined as an inconspicuous mGS according the reference centiles adjusted to sex and age, and a low mGS according the reference centiles adjusted to sex, age, height and BMI.

To investigate the relationship between the status "false positive classified as low mGS" and the sum of the z-scores for height and BMI, a logistic regression was performed (*glm* function in R). The same regression was also performed for the status “false negative classified as low mGS”. This analysis was performed for both sexes together, because of the paucity of data with extreme z-scores for height and BMI.

# Tables

| Suppl. Table 1 Reference centiles for max. grip strength (in kg) of females | | | | | | | | | | | |
| --- | --- | --- | --- | --- | --- | --- | --- | --- | --- | --- | --- |
| Age, years | C1 | C3 | C10 | C25 | C50 | C75 | C90 | C97 | C99 | S | L |
| 6.0 | 5.6 | 6.3 | 7.3 | 8.4 | 9.8 | 11.2 | 12.5 | 13.9 | 15.0 | 0.207 | 0.530 |
| 6.5 | 6.2 | 7.0 | 8.1 | 9.3 | 10.8 | 12.3 | 13.8 | 15.3 | 16.4 | 0.204 | 0.538 |
| 7.0 | 6.8 | 7.7 | 8.9 | 10.2 | 11.8 | 13.4 | 15.0 | 16.6 | 17.8 | 0.201 | 0.546 |
| 7.5 | 7.5 | 8.4 | 9.7 | 11.1 | 12.7 | 14.5 | 16.2 | 17.9 | 19.2 | 0.199 | 0.554 |
| 8.0 | 8.1 | 9.1 | 10.5 | 11.9 | 13.7 | 15.6 | 17.3 | 19.1 | 20.6 | 0.196 | 0.563 |
| 8.5 | 8.7 | 9.7 | 11.2 | 12.8 | 14.7 | 16.6 | 18.5 | 20.4 | 21.9 | 0.193 | 0.571 |
| 9.0 | 9.3 | 10.4 | 12.0 | 13.7 | 15.6 | 17.7 | 19.6 | 21.6 | 23.2 | 0.191 | 0.580 |
| 9.5 | 10.0 | 11.2 | 12.8 | 14.5 | 16.6 | 18.8 | 20.8 | 22.9 | 24.5 | 0.188 | 0.588 |
| 10.0 | 10.7 | 11.9 | 13.6 | 15.5 | 17.6 | 19.9 | 22.0 | 24.2 | 25.9 | 0.186 | 0.596 |
| 10.5 | 11.4 | 12.7 | 14.5 | 16.4 | 18.7 | 21.1 | 23.3 | 25.6 | 27.4 | 0.184 | 0.603 |
| 11.0 | 12.2 | 13.5 | 15.4 | 17.5 | 19.8 | 22.3 | 24.7 | 27.1 | 28.9 | 0.182 | 0.608 |
| 11.5 | 13.0 | 14.4 | 16.4 | 18.5 | 21.0 | 23.6 | 26.1 | 28.6 | 30.5 | 0.180 | 0.613 |
| 12.0 | 13.8 | 15.2 | 17.3 | 19.6 | 22.2 | 24.9 | 27.5 | 30.1 | 32.1 | 0.178 | 0.616 |
| 12.5 | 14.6 | 16.1 | 18.3 | 20.6 | 23.3 | 26.2 | 28.8 | 31.5 | 33.6 | 0.176 | 0.617 |
| 13.0 | 15.3 | 17.0 | 19.2 | 21.6 | 24.4 | 27.4 | 30.1 | 32.9 | 35.1 | 0.174 | 0.615 |
| 13.5 | 16.1 | 17.8 | 20.1 | 22.6 | 25.5 | 28.5 | 31.3 | 34.2 | 36.5 | 0.172 | 0.611 |
| 14.0 | 16.8 | 18.5 | 20.9 | 23.4 | 26.4 | 29.5 | 32.4 | 35.4 | 37.7 | 0.171 | 0.604 |
| 14.5 | 17.4 | 19.2 | 21.6 | 24.2 | 27.2 | 30.4 | 33.4 | 36.5 | 38.8 | 0.169 | 0.594 |
| 15.0 | 18.0 | 19.8 | 22.2 | 24.9 | 28.0 | 31.2 | 34.2 | 37.3 | 39.7 | 0.167 | 0.580 |
| 15.5 | 18.5 | 20.3 | 22.8 | 25.5 | 28.6 | 31.8 | 34.9 | 38.1 | 40.5 | 0.166 | 0.563 |
| 16.0 | 18.9 | 20.7 | 23.2 | 25.9 | 29.1 | 32.4 | 35.5 | 38.7 | 41.1 | 0.164 | 0.542 |
| 16.5 | 19.3 | 21.1 | 23.6 | 26.3 | 29.5 | 32.8 | 35.9 | 39.2 | 41.6 | 0.163 | 0.518 |
| 17.0 | 19.7 | 21.4 | 23.9 | 26.6 | 29.8 | 33.1 | 36.3 | 39.5 | 42.0 | 0.161 | 0.492 |
| 17.5 | 19.9 | 21.7 | 24.2 | 26.9 | 30.0 | 33.4 | 36.5 | 39.8 | 42.3 | 0.160 | 0.462 |
| 18.0 | 20.2 | 22.0 | 24.4 | 27.1 | 30.2 | 33.6 | 36.7 | 40.0 | 42.6 | 0.159 | 0.431 |
| 18.5 | 20.5 | 22.2 | 24.6 | 27.3 | 30.4 | 33.7 | 36.9 | 40.3 | 42.8 | 0.158 | 0.397 |
| 19.0 | 20.7 | 22.4 | 24.8 | 27.5 | 30.6 | 33.9 | 37.1 | 40.4 | 43.1 | 0.157 | 0.361 |
| 19.5 | 20.9 | 22.6 | 25.0 | 27.6 | 30.7 | 34.1 | 37.3 | 40.6 | 43.3 | 0.155 | 0.324 |
| 20.0 | 21.1 | 22.8 | 25.2 | 27.8 | 30.9 | 34.2 | 37.4 | 40.8 | 43.5 | 0.154 | 0.285 |
| 20.5 | 21.3 | 23.0 | 25.4 | 27.9 | 31.0 | 34.3 | 37.6 | 41.0 | 43.6 | 0.153 | 0.246 |
| 21.0 | 21.5 | 23.2 | 25.5 | 28.1 | 31.1 | 34.5 | 37.7 | 41.1 | 43.8 | 0.152 | 0.206 |
| 21.5 | 21.7 | 23.3 | 25.6 | 28.2 | 31.2 | 34.5 | 37.8 | 41.2 | 44.0 | 0.151 | 0.166 |
| 22.0 | 21.9 | 23.5 | 25.8 | 28.3 | 31.3 | 34.6 | 37.9 | 41.3 | 44.1 | 0.150 | 0.125 |
| 22.5 | 22.1 | 23.6 | 25.9 | 28.4 | 31.4 | 34.7 | 38.0 | 41.4 | 44.2 | 0.149 | 0.085 |
| 23.0 | 22.2 | 23.8 | 26.0 | 28.5 | 31.5 | 34.8 | 38.0 | 41.5 | 44.3 | 0.148 | 0.045 |
| 23.5 | 22.4 | 23.9 | 26.1 | 28.6 | 31.6 | 34.9 | 38.1 | 41.6 | 44.4 | 0.147 | 0.005 |
| 24.0 | 22.6 | 24.1 | 26.3 | 28.7 | 31.6 | 34.9 | 38.2 | 41.7 | 44.5 | 0.146 | -0.036 |

| Suppl. Table 2 Reference centiles for max. grip strength (in kg) for males | | | | | | | | | | | |
| --- | --- | --- | --- | --- | --- | --- | --- | --- | --- | --- | --- |
| Age, years | C1 | C3 | C10 | C25 | C50 | C75 | C90 | C97 | C99 | S | L |
| 6.0 | 6.0 | 6.8 | 7.8 | 9.0 | 10.4 | 11.9 | 13.3 | 14.7 | 15.9 | 0.204 | 0.527 |
| 6.5 | 6.7 | 7.5 | 8.7 | 10.0 | 11.5 | 13.1 | 14.7 | 16.3 | 17.5 | 0.203 | 0.508 |
| 7.0 | 7.4 | 8.2 | 9.5 | 10.9 | 12.5 | 14.3 | 16.0 | 17.7 | 19.1 | 0.201 | 0.490 |
| 7.5 | 8.0 | 8.9 | 10.3 | 11.8 | 13.5 | 15.4 | 17.2 | 19.1 | 20.6 | 0.200 | 0.472 |
| 8.0 | 8.6 | 9.6 | 11.0 | 12.6 | 14.5 | 16.5 | 18.4 | 20.5 | 22.0 | 0.199 | 0.454 |
| 8.5 | 9.2 | 10.3 | 11.8 | 13.4 | 15.4 | 17.5 | 19.6 | 21.7 | 23.4 | 0.198 | 0.436 |
| 9.0 | 9.8 | 10.9 | 12.5 | 14.2 | 16.3 | 18.6 | 20.7 | 23.0 | 24.8 | 0.196 | 0.419 |
| 9.5 | 10.4 | 11.6 | 13.2 | 15.0 | 17.2 | 19.6 | 21.8 | 24.2 | 26.1 | 0.195 | 0.404 |
| 10.0 | 11.0 | 12.2 | 13.9 | 15.8 | 18.1 | 20.6 | 23.0 | 25.5 | 27.5 | 0.195 | 0.392 |
| 10.5 | 11.6 | 12.9 | 14.7 | 16.7 | 19.1 | 21.7 | 24.2 | 26.8 | 28.9 | 0.194 | 0.382 |
| 11.0 | 12.3 | 13.6 | 15.5 | 17.6 | 20.1 | 22.8 | 25.5 | 28.3 | 30.4 | 0.193 | 0.375 |
| 11.5 | 13.1 | 14.4 | 16.4 | 18.7 | 21.3 | 24.2 | 27.0 | 29.9 | 32.2 | 0.192 | 0.372 |
| 12.0 | 14.0 | 15.4 | 17.6 | 19.9 | 22.7 | 25.8 | 28.8 | 31.9 | 34.4 | 0.192 | 0.373 |
| 12.5 | 15.0 | 16.6 | 18.9 | 21.4 | 24.5 | 27.8 | 31.0 | 34.3 | 36.9 | 0.191 | 0.380 |
| 13.0 | 16.3 | 18.0 | 20.5 | 23.2 | 26.5 | 30.0 | 33.4 | 37.0 | 39.9 | 0.191 | 0.392 |
| 13.5 | 17.6 | 19.5 | 22.2 | 25.1 | 28.7 | 32.5 | 36.1 | 40.0 | 43.0 | 0.190 | 0.409 |
| 14.0 | 19.0 | 21.0 | 24.0 | 27.1 | 31.0 | 35.0 | 39.0 | 43.1 | 46.3 | 0.189 | 0.432 |
| 14.5 | 20.4 | 22.6 | 25.8 | 29.2 | 33.3 | 37.7 | 41.9 | 46.2 | 49.6 | 0.188 | 0.460 |
| 15.0 | 21.8 | 24.2 | 27.6 | 31.3 | 35.6 | 40.3 | 44.7 | 49.3 | 52.9 | 0.187 | 0.492 |
| 15.5 | 23.2 | 25.8 | 29.4 | 33.3 | 37.9 | 42.8 | 47.4 | 52.2 | 56.0 | 0.186 | 0.528 |
| 16.0 | 24.5 | 27.2 | 31.0 | 35.2 | 40.0 | 45.1 | 49.9 | 54.9 | 58.7 | 0.184 | 0.569 |
| 16.5 | 25.6 | 28.5 | 32.5 | 36.9 | 41.9 | 47.2 | 52.2 | 57.2 | 61.1 | 0.183 | 0.614 |
| 17.0 | 26.6 | 29.6 | 33.9 | 38.4 | 43.6 | 49.0 | 54.1 | 59.2 | 63.2 | 0.181 | 0.662 |
| 17.5 | 27.4 | 30.6 | 35.0 | 39.7 | 45.0 | 50.6 | 55.7 | 60.9 | 64.9 | 0.179 | 0.712 |
| 18.0 | 28.2 | 31.5 | 36.0 | 40.8 | 46.3 | 51.9 | 57.1 | 62.3 | 66.3 | 0.178 | 0.765 |
| 18.5 | 28.8 | 32.2 | 36.9 | 41.8 | 47.3 | 53.0 | 58.2 | 63.4 | 67.3 | 0.176 | 0.819 |
| 19.0 | 29.2 | 32.8 | 37.6 | 42.6 | 48.2 | 53.9 | 59.1 | 64.3 | 68.2 | 0.174 | 0.875 |
| 19.5 | 29.6 | 33.2 | 38.2 | 43.2 | 48.9 | 54.6 | 59.8 | 64.9 | 68.8 | 0.172 | 0.930 |
| 20.0 | 29.9 | 33.6 | 38.7 | 43.8 | 49.5 | 55.2 | 60.3 | 65.4 | 69.2 | 0.171 | 0.987 |
| 20.5 | 30.1 | 33.9 | 39.0 | 44.2 | 49.9 | 55.6 | 60.7 | 65.7 | 69.4 | 0.169 | 1.043 |
| 21.0 | 30.2 | 34.2 | 39.4 | 44.5 | 50.3 | 55.9 | 60.9 | 65.9 | 69.5 | 0.167 | 1.099 |
| 21.5 | 30.3 | 34.3 | 39.6 | 44.8 | 50.5 | 56.1 | 61.1 | 65.9 | 69.5 | 0.166 | 1.154 |
| 22.0 | 30.4 | 34.5 | 39.8 | 45.0 | 50.7 | 56.3 | 61.2 | 65.9 | 69.4 | 0.164 | 1.210 |
| 22.5 | 30.4 | 34.6 | 39.9 | 45.2 | 50.9 | 56.4 | 61.2 | 65.8 | 69.3 | 0.163 | 1.267 |
| 23.0 | 30.4 | 34.6 | 40.1 | 45.4 | 51.0 | 56.4 | 61.2 | 65.8 | 69.1 | 0.161 | 1.323 |
| 23.5 | 30.4 | 34.7 | 40.2 | 45.5 | 51.1 | 56.5 | 61.2 | 65.7 | 69.0 | 0.159 | 1.379 |
| 24.0 | 30.4 | 34.8 | 40.3 | 45.6 | 51.2 | 56.6 | 61.2 | 65.6 | 68.8 | 0.158 | 1.435 |

| Suppl. Table 3 Reference centiles for max. grip strength (in kg) for females, adjusted for predicted grip strength (pGS) | | | | | | | | | | | |
| --- | --- | --- | --- | --- | --- | --- | --- | --- | --- | --- | --- |
| pGS, kg | C1 | C3 | C10 | C25 | C50 | C75 | C90 | C97 | C99 | S | L |
| 7 | 4.8 | 5.3 | 6.0 | 6.7 | 7.7 | 8.8 | 9.9 | 11.1 | 12.1 | 0.198 | 0.074 |
| 8 | 5.4 | 5.9 | 6.7 | 7.5 | 8.5 | 9.7 | 10.9 | 12.2 | 13.2 | 0.192 | 0.134 |
| 9 | 6.0 | 6.5 | 7.4 | 8.3 | 9.4 | 10.6 | 11.9 | 13.2 | 14.3 | 0.187 | 0.193 |
| 10 | 6.6 | 7.2 | 8.1 | 9.1 | 10.3 | 11.6 | 12.9 | 14.2 | 15.3 | 0.181 | 0.249 |
| 11 | 7.2 | 7.9 | 8.8 | 9.9 | 11.1 | 12.5 | 13.9 | 15.3 | 16.4 | 0.176 | 0.304 |
| 12 | 7.8 | 8.5 | 9.6 | 10.7 | 12.0 | 13.5 | 14.9 | 16.3 | 17.5 | 0.172 | 0.356 |
| 13 | 8.4 | 9.2 | 10.3 | 11.5 | 12.9 | 14.4 | 15.9 | 17.4 | 18.5 | 0.167 | 0.408 |
| 14 | 9.1 | 9.9 | 11.0 | 12.3 | 13.8 | 15.3 | 16.8 | 18.4 | 19.6 | 0.163 | 0.457 |
| 15 | 9.7 | 10.6 | 11.8 | 13.1 | 14.6 | 16.3 | 17.8 | 19.4 | 20.6 | 0.160 | 0.503 |
| 16 | 10.3 | 11.2 | 12.5 | 13.9 | 15.5 | 17.2 | 18.7 | 20.4 | 21.6 | 0.157 | 0.544 |
| 17 | 10.9 | 11.9 | 13.3 | 14.7 | 16.4 | 18.1 | 19.7 | 21.4 | 22.6 | 0.154 | 0.578 |
| 18 | 11.6 | 12.6 | 14.0 | 15.5 | 17.2 | 19.0 | 20.7 | 22.4 | 23.7 | 0.152 | 0.606 |
| 19 | 12.3 | 13.3 | 14.8 | 16.4 | 18.2 | 20.0 | 21.8 | 23.5 | 24.9 | 0.150 | 0.629 |
| 20 | 13.0 | 14.1 | 15.6 | 17.3 | 19.1 | 21.1 | 22.9 | 24.7 | 26.1 | 0.148 | 0.647 |
| 21 | 13.7 | 14.9 | 16.5 | 18.2 | 20.2 | 22.2 | 24.1 | 26.0 | 27.5 | 0.147 | 0.663 |
| 22 | 14.5 | 15.7 | 17.5 | 19.3 | 21.3 | 23.4 | 25.4 | 27.4 | 28.9 | 0.146 | 0.678 |
| 23 | 15.3 | 16.6 | 18.4 | 20.3 | 22.5 | 24.7 | 26.8 | 28.9 | 30.4 | 0.145 | 0.692 |
| 24 | 16.2 | 17.6 | 19.5 | 21.4 | 23.7 | 26.0 | 28.2 | 30.4 | 32.0 | 0.144 | 0.707 |
| 25 | 17.0 | 18.5 | 20.5 | 22.6 | 24.9 | 27.4 | 29.6 | 31.9 | 33.6 | 0.143 | 0.723 |
| 26 | 17.9 | 19.4 | 21.5 | 23.7 | 26.1 | 28.7 | 31.0 | 33.4 | 35.2 | 0.143 | 0.742 |
| 27 | 18.6 | 20.2 | 22.4 | 24.7 | 27.3 | 29.9 | 32.4 | 34.8 | 36.7 | 0.142 | 0.763 |
| 28 | 19.3 | 21.0 | 23.3 | 25.7 | 28.4 | 31.1 | 33.6 | 36.1 | 38.0 | 0.142 | 0.788 |
| 29 | 20.0 | 21.7 | 24.1 | 26.5 | 29.3 | 32.2 | 34.7 | 37.3 | 39.3 | 0.142 | 0.819 |
| 30 | 20.5 | 22.3 | 24.8 | 27.4 | 30.2 | 33.2 | 35.8 | 38.5 | 40.4 | 0.142 | 0.856 |
| 31 | 21.0 | 22.9 | 25.5 | 28.2 | 31.1 | 34.1 | 36.9 | 39.6 | 41.6 | 0.142 | 0.900 |
| 32 | 21.5 | 23.5 | 26.2 | 29.0 | 32.0 | 35.1 | 37.9 | 40.6 | 42.7 | 0.142 | 0.950 |
| 33 | 22.0 | 24.1 | 26.9 | 29.8 | 32.9 | 36.1 | 38.9 | 41.7 | 43.8 | 0.142 | 1.004 |
| 34 | 22.4 | 24.6 | 27.6 | 30.5 | 33.8 | 37.1 | 40.0 | 42.8 | 44.9 | 0.143 | 1.061 |
| 35 | 22.9 | 25.2 | 28.3 | 31.3 | 34.7 | 38.0 | 41.0 | 43.9 | 46.0 | 0.143 | 1.119 |
| 36 | 23.3 | 25.7 | 28.9 | 32.1 | 35.6 | 39.0 | 42.0 | 45.0 | 47.2 | 0.143 | 1.178 |
| 37 | 23.7 | 26.3 | 29.6 | 32.9 | 36.5 | 40.0 | 43.1 | 46.1 | 48.3 | 0.144 | 1.238 |
| 38 | 24.1 | 26.8 | 30.3 | 33.7 | 37.4 | 41.0 | 44.1 | 47.2 | 49.4 | 0.144 | 1.297 |
| 39 | 24.5 | 27.3 | 30.9 | 34.5 | 38.3 | 42.0 | 45.2 | 48.2 | 50.5 | 0.144 | 1.357 |
| 40 | 24.8 | 27.8 | 31.6 | 35.3 | 39.2 | 42.9 | 46.2 | 49.3 | 51.6 | 0.145 | 1.417 |

| Suppl. Table 4 Reference centiles for max. grip strength (in kg) for males, adjusted for predicted grip strength (pGS) | | | | | | | | | | | |
| --- | --- | --- | --- | --- | --- | --- | --- | --- | --- | --- | --- |
| pGS, kg | C1 | C3 | C10 | C25 | C50 | C75 | C90 | C97 | C99 | S | L |
| 7 | 6.2 | 6.8 | 7.7 | 8.7 | 9.9 | 11.2 | 12.5 | 13.9 | 14.9 | 0.188 | 0.359 |
| 8 | 6.6 | 7.3 | 8.3 | 9.3 | 10.6 | 12.0 | 13.3 | 14.7 | 15.8 | 0.186 | 0.396 |
| 9 | 7.0 | 7.7 | 8.8 | 9.9 | 11.3 | 12.7 | 14.1 | 15.5 | 16.7 | 0.184 | 0.434 |
| 10 | 7.4 | 8.2 | 9.3 | 10.5 | 11.9 | 13.4 | 14.9 | 16.4 | 17.5 | 0.182 | 0.472 |
| 11 | 7.8 | 8.7 | 9.8 | 11.1 | 12.6 | 14.1 | 15.6 | 17.2 | 18.3 | 0.180 | 0.509 |
| 12 | 8.3 | 9.1 | 10.4 | 11.7 | 13.2 | 14.8 | 16.4 | 18.0 | 19.2 | 0.178 | 0.546 |
| 13 | 8.7 | 9.6 | 10.9 | 12.3 | 13.9 | 15.6 | 17.1 | 18.8 | 20.0 | 0.176 | 0.582 |
| 14 | 9.1 | 10.1 | 11.4 | 12.8 | 14.5 | 16.3 | 17.9 | 19.6 | 20.8 | 0.174 | 0.618 |
| 15 | 9.5 | 10.5 | 11.9 | 13.4 | 15.2 | 17.0 | 18.6 | 20.4 | 21.7 | 0.173 | 0.652 |
| 16 | 9.9 | 11.0 | 12.5 | 14.0 | 15.8 | 17.7 | 19.4 | 21.1 | 22.5 | 0.171 | 0.686 |
| 17 | 10.3 | 11.4 | 13.0 | 14.6 | 16.4 | 18.3 | 20.1 | 21.9 | 23.3 | 0.170 | 0.718 |
| 18 | 10.7 | 11.9 | 13.5 | 15.1 | 17.0 | 19.0 | 20.8 | 22.6 | 24.0 | 0.168 | 0.749 |
| 19 | 11.1 | 12.3 | 14.0 | 15.7 | 17.6 | 19.6 | 21.5 | 23.3 | 24.8 | 0.167 | 0.779 |
| 20 | 11.5 | 12.7 | 14.4 | 16.2 | 18.2 | 20.3 | 22.1 | 24.0 | 25.5 | 0.165 | 0.808 |
| 21 | 11.8 | 13.1 | 14.9 | 16.7 | 18.8 | 20.9 | 22.8 | 24.7 | 26.2 | 0.164 | 0.836 |
| 22 | 12.2 | 13.5 | 15.4 | 17.2 | 19.4 | 21.5 | 23.5 | 25.4 | 26.9 | 0.163 | 0.862 |
| 23 | 12.6 | 14.0 | 15.9 | 17.8 | 20.0 | 22.2 | 24.2 | 26.2 | 27.6 | 0.162 | 0.886 |
| 24 | 13.0 | 14.4 | 16.4 | 18.4 | 20.6 | 22.9 | 24.9 | 26.9 | 28.5 | 0.162 | 0.908 |
| 25 | 13.4 | 14.9 | 16.9 | 19.0 | 21.3 | 23.6 | 25.7 | 27.8 | 29.4 | 0.161 | 0.928 |
| 26 | 13.9 | 15.5 | 17.6 | 19.7 | 22.1 | 24.5 | 26.6 | 28.8 | 30.4 | 0.160 | 0.946 |
| 27 | 14.5 | 16.1 | 18.2 | 20.5 | 22.9 | 25.4 | 27.6 | 29.9 | 31.5 | 0.160 | 0.963 |
| 28 | 15.1 | 16.8 | 19.0 | 21.3 | 23.9 | 26.5 | 28.8 | 31.1 | 32.8 | 0.160 | 0.978 |
| 29 | 15.8 | 17.5 | 19.9 | 22.3 | 25.0 | 27.7 | 30.1 | 32.5 | 34.3 | 0.159 | 0.992 |
| 30 | 16.5 | 18.4 | 20.9 | 23.4 | 26.3 | 29.1 | 31.6 | 34.1 | 36.0 | 0.159 | 1.005 |
| 31 | 17.3 | 19.3 | 22.0 | 24.6 | 27.6 | 30.6 | 33.2 | 35.9 | 37.8 | 0.159 | 1.016 |
| 32 | 18.2 | 20.3 | 23.1 | 25.9 | 29.1 | 32.2 | 35.0 | 37.7 | 39.8 | 0.159 | 1.027 |
| 33 | 19.2 | 21.4 | 24.3 | 27.3 | 30.6 | 33.9 | 36.8 | 39.7 | 41.8 | 0.159 | 1.037 |
| 34 | 20.1 | 22.4 | 25.6 | 28.7 | 32.2 | 35.6 | 38.7 | 41.7 | 44.0 | 0.159 | 1.047 |
| 35 | 21.1 | 23.5 | 26.8 | 30.1 | 33.8 | 37.4 | 40.6 | 43.8 | 46.1 | 0.159 | 1.055 |
| 36 | 22.1 | 24.7 | 28.1 | 31.5 | 35.4 | 39.1 | 42.5 | 45.9 | 48.3 | 0.159 | 1.064 |
| 37 | 23.1 | 25.8 | 29.4 | 33.0 | 37.0 | 40.9 | 44.5 | 47.9 | 50.5 | 0.159 | 1.072 |
| 38 | 24.0 | 26.8 | 30.6 | 34.4 | 38.5 | 42.7 | 46.4 | 50.0 | 52.6 | 0.159 | 1.079 |
| 39 | 24.9 | 27.9 | 31.8 | 35.8 | 40.1 | 44.4 | 48.2 | 52.0 | 54.7 | 0.159 | 1.086 |
| 40 | 25.8 | 28.9 | 33.0 | 37.1 | 41.6 | 46.0 | 50.0 | 53.8 | 56.7 | 0.159 | 1.094 |
| 41 | 26.7 | 29.9 | 34.1 | 38.3 | 42.9 | 47.5 | 51.6 | 55.6 | 58.6 | 0.159 | 1.101 |
| 42 | 27.5 | 30.7 | 35.1 | 39.5 | 44.2 | 49.0 | 53.2 | 57.3 | 60.3 | 0.159 | 1.109 |
| 43 | 28.2 | 31.6 | 36.0 | 40.5 | 45.4 | 50.3 | 54.6 | 58.8 | 61.9 | 0.159 | 1.117 |
| 44 | 28.8 | 32.3 | 36.9 | 41.5 | 46.5 | 51.5 | 55.9 | 60.2 | 63.4 | 0.159 | 1.125 |
| 45 | 29.4 | 33.0 | 37.7 | 42.4 | 47.5 | 52.6 | 57.1 | 61.5 | 64.7 | 0.159 | 1.133 |
| 46 | 30.0 | 33.6 | 38.4 | 43.2 | 48.5 | 53.6 | 58.2 | 62.7 | 66.0 | 0.159 | 1.142 |
| 47 | 30.5 | 34.2 | 39.1 | 44.0 | 49.3 | 54.6 | 59.2 | 63.7 | 67.1 | 0.159 | 1.151 |
| 48 | 30.9 | 34.7 | 39.7 | 44.7 | 50.1 | 55.4 | 60.1 | 64.7 | 68.1 | 0.159 | 1.160 |
| 49 | 31.4 | 35.2 | 40.3 | 45.4 | 50.8 | 56.2 | 61.0 | 65.7 | 69.1 | 0.159 | 1.169 |
| 50 | 31.8 | 35.7 | 40.9 | 46.0 | 51.6 | 57.0 | 61.9 | 66.6 | 70.0 | 0.158 | 1.178 |
| 51 | 32.2 | 36.2 | 41.4 | 46.6 | 52.3 | 57.8 | 62.7 | 67.4 | 70.9 | 0.158 | 1.187 |
| 52 | 32.6 | 36.6 | 42.0 | 47.2 | 52.9 | 58.5 | 63.5 | 68.3 | 71.8 | 0.158 | 1.196 |
| 53 | 33.0 | 37.1 | 42.5 | 47.8 | 53.6 | 59.2 | 64.2 | 69.1 | 72.7 | 0.158 | 1.205 |
| 54 | 33.4 | 37.5 | 43.0 | 48.4 | 54.3 | 60.0 | 65.0 | 69.9 | 73.5 | 0.158 | 1.215 |
| 55 | 33.8 | 38.0 | 43.5 | 49.0 | 54.9 | 60.7 | 65.8 | 70.7 | 74.4 | 0.158 | 1.224 |
| 56 | 34.1 | 38.4 | 44.1 | 49.6 | 55.6 | 61.4 | 66.6 | 71.5 | 75.2 | 0.158 | 1.233 |

| Suppl. Table 5 Results of the Q-Permutation test for normality of the z-scores for mGS, adjusted for sex, age, height and BMI | | | | | | |  |
| --- | --- | --- | --- | --- | --- | --- | --- |
|  |  |  |  |  |  |  |  |
|  |  |  |  |  |  |  |  |
| Explanatory | Females | | | Males | | |  |
| variable | Q1 | Q2 | Q3 | Q1 | Q2 | Q3 |  |
| Age, p-value | 0.596 | 0.411 | 0.596 | 0.508 | 0.724 | 0.330 |  |
| height, p-value | 0.292 | 0.193 | 0.578 | 0.889 | 0.214 | 0.269 |  |
| BMI, p-value | 0.156 | 0.605 | 0.516 | 0.929 | 0.043 | 0.316 |  |
| Q-Permutation tests were performed with n=5000 permutations and n=9 intervals of the explanatory variables. P-values < 0.05 indicate a significant deviation from normality assumption. Q1 statistics assess the median, Q2 the standard deviation and Q3 the skewness of the distribution. More details are given in section “Statistical analysis”. | | | | | | |  |
|  |  |  |  |  |  |  |  |
|  |  |  |  |  |  |  |  |
|  |  |  |  |  |  |  |  |

| Suppl. Table 6 Reference centiles for annualized differences in z-scores for max. grip strength in females, adjusted to sex and age | | | | | | | | | | | |
| --- | --- | --- | --- | --- | --- | --- | --- | --- | --- | --- | --- |
|  |  |  |  |  |  |  |  |  |  |  |  |
| Age, years | C1 | C3 | C10 | C25 | C50 | C75 | C90 | C97 | C99 | S | L |
| 6.0 | -0.94 | -0.71 | -0.42 | -0.16 | 0.11 | 0.37 | 0.63 | 0.92 | 1.15 | 0.422 | 1.578 |
| 6.5 | -0.93 | -0.71 | -0.43 | -0.17 | 0.09 | 0.35 | 0.61 | 0.89 | 1.11 | 0.415 | 1.581 |
| 7.0 | -0.93 | -0.71 | -0.43 | -0.18 | 0.08 | 0.33 | 0.58 | 0.86 | 1.08 | 0.407 | 1.583 |
| 7.5 | -0.93 | -0.71 | -0.44 | -0.19 | 0.06 | 0.31 | 0.56 | 0.83 | 1.05 | 0.400 | 1.587 |
| 8.0 | -0.92 | -0.71 | -0.44 | -0.20 | 0.05 | 0.29 | 0.54 | 0.81 | 1.02 | 0.393 | 1.593 |
| 8.5 | -0.91 | -0.70 | -0.44 | -0.20 | 0.04 | 0.28 | 0.52 | 0.78 | 0.99 | 0.387 | 1.602 |
| 9.0 | -0.90 | -0.70 | -0.44 | -0.20 | 0.04 | 0.27 | 0.51 | 0.77 | 0.97 | 0.381 | 1.613 |
| 9.5 | -0.88 | -0.68 | -0.43 | -0.20 | 0.04 | 0.27 | 0.51 | 0.76 | 0.96 | 0.375 | 1.627 |
| 10.0 | -0.85 | -0.66 | -0.42 | -0.19 | 0.05 | 0.28 | 0.51 | 0.76 | 0.95 | 0.370 | 1.646 |
| 10.5 | -0.83 | -0.64 | -0.40 | -0.17 | 0.06 | 0.29 | 0.52 | 0.76 | 0.95 | 0.365 | 1.672 |
| 11.0 | -0.80 | -0.62 | -0.38 | -0.16 | 0.07 | 0.30 | 0.52 | 0.76 | 0.94 | 0.360 | 1.704 |
| 11.5 | -0.77 | -0.60 | -0.37 | -0.15 | 0.08 | 0.31 | 0.53 | 0.75 | 0.93 | 0.354 | 1.737 |
| 12.0 | -0.75 | -0.58 | -0.36 | -0.14 | 0.08 | 0.31 | 0.52 | 0.75 | 0.92 | 0.349 | 1.769 |
| 12.5 | -0.74 | -0.57 | -0.35 | -0.14 | 0.08 | 0.31 | 0.52 | 0.74 | 0.90 | 0.343 | 1.796 |
| 13.0 | -0.73 | -0.56 | -0.35 | -0.14 | 0.08 | 0.30 | 0.51 | 0.72 | 0.88 | 0.337 | 1.812 |
| 13.5 | -0.72 | -0.56 | -0.35 | -0.15 | 0.07 | 0.29 | 0.49 | 0.70 | 0.86 | 0.331 | 1.813 |
| 14.0 | -0.71 | -0.56 | -0.35 | -0.15 | 0.06 | 0.27 | 0.47 | 0.68 | 0.83 | 0.324 | 1.806 |
| 14.5 | -0.71 | -0.56 | -0.36 | -0.16 | 0.05 | 0.25 | 0.45 | 0.65 | 0.80 | 0.317 | 1.797 |
| 15.0 | -0.71 | -0.56 | -0.36 | -0.17 | 0.03 | 0.24 | 0.43 | 0.62 | 0.77 | 0.310 | 1.791 |
| 15.5 | -0.70 | -0.55 | -0.36 | -0.17 | 0.02 | 0.22 | 0.40 | 0.60 | 0.74 | 0.302 | 1.790 |
| 16.0 | -0.69 | -0.55 | -0.36 | -0.18 | 0.01 | 0.20 | 0.38 | 0.57 | 0.71 | 0.294 | 1.791 |
| 16.5 | -0.68 | -0.54 | -0.36 | -0.18 | 0.00 | 0.19 | 0.36 | 0.55 | 0.68 | 0.285 | 1.790 |
| 17.0 | -0.66 | -0.53 | -0.35 | -0.18 | 0.00 | 0.18 | 0.35 | 0.53 | 0.66 | 0.277 | 1.783 |
| 17.5 | -0.64 | -0.51 | -0.34 | -0.17 | 0.00 | 0.17 | 0.34 | 0.51 | 0.64 | 0.268 | 1.766 |
| 18.0 | -0.61 | -0.49 | -0.32 | -0.16 | 0.01 | 0.17 | 0.33 | 0.50 | 0.63 | 0.259 | 1.745 |
| 18.5 | -0.58 | -0.46 | -0.30 | -0.14 | 0.02 | 0.18 | 0.33 | 0.50 | 0.62 | 0.249 | 1.730 |
| 19.0 | -0.54 | -0.42 | -0.27 | -0.12 | 0.04 | 0.19 | 0.34 | 0.49 | 0.61 | 0.240 | 1.730 |
| 19.5 | -0.50 | -0.39 | -0.24 | -0.09 | 0.05 | 0.20 | 0.35 | 0.50 | 0.61 | 0.231 | 1.743 |
| 20.0 | -0.46 | -0.35 | -0.20 | -0.07 | 0.08 | 0.22 | 0.36 | 0.50 | 0.61 | 0.223 | 1.769 |

| Suppl. Table 7 Reference centiles for annualized differences in z-scores for max. grip strength in males, adjusted to sex and age | | | | | | | | | | | |
| --- | --- | --- | --- | --- | --- | --- | --- | --- | --- | --- | --- |
|  |  |  |  |  |  |  |  |  |  |  |  |
| Age, years | C1 | C3 | C10 | C25 | C50 | C75 | C90 | C97 | C99 | S | L |
| 6.0 | -0.73 | -0.56 | -0.33 | -0.10 | 0.14 | 0.38 | 0.60 | 0.83 | 1.01 | 0.367 | 1.835 |
| 6.5 | -0.73 | -0.55 | -0.33 | -0.11 | 0.12 | 0.36 | 0.57 | 0.80 | 0.97 | 0.356 | 1.804 |
| 7.0 | -0.72 | -0.55 | -0.33 | -0.11 | 0.11 | 0.33 | 0.55 | 0.77 | 0.94 | 0.345 | 1.774 |
| 7.5 | -0.71 | -0.55 | -0.33 | -0.12 | 0.09 | 0.31 | 0.52 | 0.73 | 0.90 | 0.335 | 1.745 |
| 8.0 | -0.71 | -0.55 | -0.33 | -0.13 | 0.08 | 0.29 | 0.49 | 0.70 | 0.86 | 0.326 | 1.723 |
| 8.5 | -0.71 | -0.55 | -0.34 | -0.15 | 0.06 | 0.26 | 0.46 | 0.67 | 0.83 | 0.318 | 1.713 |
| 9.0 | -0.72 | -0.57 | -0.36 | -0.17 | 0.03 | 0.23 | 0.43 | 0.63 | 0.79 | 0.313 | 1.714 |
| 9.5 | -0.74 | -0.59 | -0.39 | -0.19 | 0.01 | 0.20 | 0.40 | 0.60 | 0.75 | 0.310 | 1.729 |
| 10.0 | -0.77 | -0.61 | -0.41 | -0.22 | -0.02 | 0.18 | 0.37 | 0.57 | 0.72 | 0.310 | 1.753 |
| 10.5 | -0.79 | -0.64 | -0.44 | -0.24 | -0.04 | 0.16 | 0.36 | 0.56 | 0.71 | 0.313 | 1.781 |
| 11.0 | -0.81 | -0.65 | -0.45 | -0.26 | -0.05 | 0.16 | 0.36 | 0.56 | 0.71 | 0.319 | 1.802 |
| 11.5 | -0.82 | -0.66 | -0.45 | -0.25 | -0.04 | 0.18 | 0.38 | 0.59 | 0.74 | 0.327 | 1.808 |
| 12.0 | -0.82 | -0.65 | -0.44 | -0.23 | -0.01 | 0.21 | 0.41 | 0.63 | 0.79 | 0.337 | 1.800 |
| 12.5 | -0.81 | -0.64 | -0.42 | -0.21 | 0.02 | 0.24 | 0.46 | 0.68 | 0.85 | 0.347 | 1.785 |
| 13.0 | -0.80 | -0.63 | -0.40 | -0.18 | 0.05 | 0.28 | 0.50 | 0.73 | 0.90 | 0.355 | 1.766 |
| 13.5 | -0.79 | -0.61 | -0.38 | -0.16 | 0.08 | 0.31 | 0.53 | 0.76 | 0.94 | 0.361 | 1.749 |
| 14.0 | -0.78 | -0.60 | -0.37 | -0.14 | 0.09 | 0.32 | 0.55 | 0.78 | 0.96 | 0.362 | 1.738 |
| 14.5 | -0.77 | -0.59 | -0.36 | -0.14 | 0.09 | 0.33 | 0.55 | 0.78 | 0.96 | 0.360 | 1.736 |
| 15.0 | -0.77 | -0.59 | -0.36 | -0.15 | 0.08 | 0.31 | 0.53 | 0.76 | 0.93 | 0.353 | 1.737 |
| 15.5 | -0.77 | -0.60 | -0.38 | -0.16 | 0.06 | 0.28 | 0.49 | 0.71 | 0.88 | 0.342 | 1.734 |
| 16.0 | -0.77 | -0.61 | -0.39 | -0.19 | 0.02 | 0.23 | 0.44 | 0.65 | 0.81 | 0.328 | 1.728 |
| 16.5 | -0.77 | -0.61 | -0.41 | -0.21 | -0.01 | 0.19 | 0.38 | 0.58 | 0.74 | 0.312 | 1.721 |
| 17.0 | -0.75 | -0.60 | -0.41 | -0.23 | -0.04 | 0.15 | 0.33 | 0.52 | 0.67 | 0.294 | 1.710 |
| 17.5 | -0.72 | -0.58 | -0.40 | -0.23 | -0.05 | 0.12 | 0.29 | 0.47 | 0.61 | 0.274 | 1.696 |
| 18.0 | -0.67 | -0.54 | -0.37 | -0.22 | -0.05 | 0.11 | 0.27 | 0.43 | 0.56 | 0.255 | 1.677 |
| 18.5 | -0.62 | -0.50 | -0.34 | -0.20 | -0.05 | 0.10 | 0.25 | 0.40 | 0.53 | 0.236 | 1.656 |
| 19.0 | -0.57 | -0.45 | -0.31 | -0.17 | -0.04 | 0.10 | 0.24 | 0.38 | 0.50 | 0.218 | 1.632 |
| 19.5 | -0.51 | -0.41 | -0.27 | -0.14 | -0.02 | 0.11 | 0.23 | 0.37 | 0.47 | 0.201 | 1.606 |
| 20.0 | -0.46 | -0.36 | -0.23 | -0.12 | 0.00 | 0.12 | 0.23 | 0.36 | 0.46 | 0.186 | 1.581 |

| Suppl. Table 8 Reference centiles for annualized differences in z-scores for max. grip strength in females, adjusted to sex, age, height and BMI | | | | | | | | | | | |
| --- | --- | --- | --- | --- | --- | --- | --- | --- | --- | --- | --- |
|  |  |  |  |  |  |  |  |  |  |  |  |
| Age, years | C1 | C3 | C10 | C25 | C50 | C75 | C90 | C97 | C99 | S | L |
| 6.0 | -1.11 | -0.82 | -0.47 | -0.16 | 0.13 | 0.43 | 0.74 | 1.09 | 1.38 | 0.492 | 1.427 |
| 6.5 | -1.10 | -0.82 | -0.48 | -0.17 | 0.11 | 0.40 | 0.71 | 1.05 | 1.33 | 0.481 | 1.444 |
| 7.0 | -1.09 | -0.82 | -0.48 | -0.19 | 0.10 | 0.38 | 0.68 | 1.01 | 1.28 | 0.470 | 1.462 |
| 7.5 | -1.07 | -0.81 | -0.49 | -0.20 | 0.08 | 0.35 | 0.65 | 0.97 | 1.23 | 0.460 | 1.480 |
| 8.0 | -1.06 | -0.81 | -0.50 | -0.21 | 0.06 | 0.33 | 0.62 | 0.93 | 1.18 | 0.449 | 1.499 |
| 8.5 | -1.05 | -0.80 | -0.50 | -0.22 | 0.05 | 0.32 | 0.59 | 0.90 | 1.14 | 0.439 | 1.521 |
| 9.0 | -1.03 | -0.79 | -0.50 | -0.23 | 0.04 | 0.30 | 0.57 | 0.87 | 1.10 | 0.430 | 1.546 |
| 9.5 | -1.01 | -0.78 | -0.49 | -0.23 | 0.03 | 0.29 | 0.56 | 0.85 | 1.07 | 0.421 | 1.575 |
| 10.0 | -0.98 | -0.76 | -0.48 | -0.22 | 0.03 | 0.29 | 0.55 | 0.83 | 1.05 | 0.413 | 1.610 |
| 10.5 | -0.95 | -0.74 | -0.47 | -0.22 | 0.04 | 0.29 | 0.55 | 0.82 | 1.03 | 0.405 | 1.652 |
| 11.0 | -0.92 | -0.71 | -0.45 | -0.21 | 0.05 | 0.30 | 0.55 | 0.81 | 1.01 | 0.398 | 1.697 |
| 11.5 | -0.89 | -0.69 | -0.44 | -0.20 | 0.05 | 0.30 | 0.55 | 0.80 | 0.99 | 0.390 | 1.741 |
| 12.0 | -0.86 | -0.67 | -0.43 | -0.19 | 0.06 | 0.31 | 0.54 | 0.79 | 0.98 | 0.383 | 1.780 |
| 12.5 | -0.83 | -0.65 | -0.41 | -0.18 | 0.06 | 0.31 | 0.54 | 0.78 | 0.96 | 0.376 | 1.811 |
| 13.0 | -0.81 | -0.63 | -0.40 | -0.17 | 0.07 | 0.31 | 0.54 | 0.77 | 0.94 | 0.369 | 1.831 |
| 13.5 | -0.79 | -0.62 | -0.39 | -0.17 | 0.07 | 0.30 | 0.53 | 0.75 | 0.93 | 0.361 | 1.840 |
| 14.0 | -0.77 | -0.60 | -0.38 | -0.17 | 0.07 | 0.30 | 0.52 | 0.74 | 0.90 | 0.353 | 1.841 |
| 14.5 | -0.76 | -0.59 | -0.38 | -0.16 | 0.06 | 0.29 | 0.50 | 0.72 | 0.88 | 0.345 | 1.842 |
| 15.0 | -0.74 | -0.58 | -0.37 | -0.17 | 0.05 | 0.28 | 0.48 | 0.69 | 0.85 | 0.336 | 1.848 |
| 15.5 | -0.73 | -0.58 | -0.37 | -0.17 | 0.04 | 0.26 | 0.46 | 0.66 | 0.82 | 0.327 | 1.862 |
| 16.0 | -0.72 | -0.57 | -0.38 | -0.18 | 0.03 | 0.24 | 0.43 | 0.63 | 0.78 | 0.318 | 1.878 |
| 16.5 | -0.71 | -0.57 | -0.38 | -0.19 | 0.01 | 0.22 | 0.41 | 0.60 | 0.74 | 0.308 | 1.894 |
| 17.0 | -0.70 | -0.56 | -0.38 | -0.20 | 0.00 | 0.20 | 0.38 | 0.56 | 0.70 | 0.298 | 1.902 |
| 17.5 | -0.68 | -0.55 | -0.37 | -0.20 | -0.01 | 0.18 | 0.36 | 0.54 | 0.67 | 0.288 | 1.895 |
| 18.0 | -0.66 | -0.53 | -0.36 | -0.19 | -0.01 | 0.18 | 0.35 | 0.52 | 0.65 | 0.277 | 1.883 |
| 18.5 | -0.63 | -0.51 | -0.34 | -0.18 | 0.00 | 0.17 | 0.34 | 0.50 | 0.63 | 0.266 | 1.874 |
| 19.0 | -0.60 | -0.48 | -0.32 | -0.16 | 0.01 | 0.17 | 0.33 | 0.49 | 0.61 | 0.256 | 1.879 |
| 19.5 | -0.56 | -0.45 | -0.29 | -0.14 | 0.02 | 0.18 | 0.33 | 0.48 | 0.60 | 0.245 | 1.897 |
| 20.0 | -0.52 | -0.41 | -0.26 | -0.12 | 0.04 | 0.19 | 0.34 | 0.48 | 0.59 | 0.235 | 1.929 |

| Suppl. Table 9 Reference centiles for annualized differences in z-scores for max. grip strength in males, adjusted to sex, age, height and BMI | | | | | | | | | | | |
| --- | --- | --- | --- | --- | --- | --- | --- | --- | --- | --- | --- |
|  |  |  |  |  |  |  |  |  |  |  |  |
| Age, years | C1 | C3 | C10 | C25 | C50 | C75 | C90 | C97 | C99 | S | L |
| 6.0 | -0.81 | -0.61 | -0.36 | -0.11 | 0.16 | 0.44 | 0.69 | 0.94 | 1.13 | 0.403 | 47.183 |
| 6.5 | -0.80 | -0.61 | -0.37 | -0.12 | 0.15 | 0.41 | 0.66 | 0.91 | 1.10 | 0.394 | 43.447 |
| 7.0 | -0.80 | -0.61 | -0.37 | -0.13 | 0.13 | 0.39 | 0.63 | 0.87 | 1.06 | 0.384 | 40.007 |
| 7.5 | -0.80 | -0.62 | -0.38 | -0.14 | 0.11 | 0.37 | 0.60 | 0.84 | 1.02 | 0.375 | 36.827 |
| 8.0 | -0.80 | -0.62 | -0.39 | -0.16 | 0.09 | 0.34 | 0.57 | 0.81 | 0.99 | 0.367 | 33.796 |
| 8.5 | -0.80 | -0.62 | -0.39 | -0.17 | 0.08 | 0.32 | 0.55 | 0.78 | 0.96 | 0.359 | 30.760 |
| 9.0 | -0.81 | -0.63 | -0.40 | -0.18 | 0.06 | 0.30 | 0.52 | 0.75 | 0.93 | 0.352 | 27.638 |
| 9.5 | -0.81 | -0.64 | -0.41 | -0.19 | 0.05 | 0.28 | 0.50 | 0.73 | 0.91 | 0.345 | 24.440 |
| 10.0 | -0.82 | -0.64 | -0.42 | -0.20 | 0.03 | 0.27 | 0.48 | 0.71 | 0.89 | 0.340 | 21.255 |
| 10.5 | -0.83 | -0.65 | -0.42 | -0.21 | 0.02 | 0.25 | 0.47 | 0.69 | 0.88 | 0.335 | 18.167 |
| 11.0 | -0.85 | -0.66 | -0.43 | -0.21 | 0.01 | 0.24 | 0.46 | 0.69 | 0.87 | 0.331 | 15.270 |
| 11.5 | -0.86 | -0.67 | -0.43 | -0.22 | 0.01 | 0.24 | 0.45 | 0.69 | 0.88 | 0.327 | 12.670 |
| 12.0 | -0.88 | -0.67 | -0.43 | -0.22 | 0.01 | 0.24 | 0.45 | 0.69 | 0.90 | 0.323 | 10.507 |
| 12.5 | -0.90 | -0.68 | -0.43 | -0.21 | 0.01 | 0.24 | 0.45 | 0.70 | 0.92 | 0.320 | 8.812 |
| 13.0 | -0.91 | -0.68 | -0.43 | -0.21 | 0.01 | 0.24 | 0.46 | 0.71 | 0.94 | 0.316 | 7.579 |
| 13.5 | -0.93 | -0.69 | -0.43 | -0.21 | 0.01 | 0.24 | 0.46 | 0.72 | 0.96 | 0.311 | 6.762 |
| 14.0 | -0.93 | -0.69 | -0.42 | -0.20 | 0.01 | 0.23 | 0.45 | 0.72 | 0.96 | 0.306 | 6.251 |
| 14.5 | -0.93 | -0.68 | -0.42 | -0.20 | 0.01 | 0.23 | 0.44 | 0.71 | 0.96 | 0.300 | 5.955 |
| 15.0 | -0.93 | -0.68 | -0.42 | -0.20 | 0.01 | 0.22 | 0.43 | 0.69 | 0.94 | 0.293 | 5.817 |
| 15.5 | -0.91 | -0.67 | -0.42 | -0.21 | 0.00 | 0.20 | 0.41 | 0.66 | 0.91 | 0.286 | 5.805 |
| 16.0 | -0.89 | -0.66 | -0.41 | -0.21 | -0.01 | 0.19 | 0.39 | 0.63 | 0.87 | 0.279 | 5.893 |
| 16.5 | -0.87 | -0.64 | -0.41 | -0.21 | -0.02 | 0.17 | 0.37 | 0.60 | 0.83 | 0.270 | 6.057 |
| 17.0 | -0.83 | -0.62 | -0.40 | -0.21 | -0.03 | 0.16 | 0.35 | 0.57 | 0.78 | 0.261 | 6.314 |
| 17.5 | -0.79 | -0.59 | -0.38 | -0.20 | -0.02 | 0.16 | 0.33 | 0.55 | 0.74 | 0.252 | 6.682 |
| 18.0 | -0.74 | -0.56 | -0.36 | -0.19 | -0.02 | 0.15 | 0.32 | 0.52 | 0.71 | 0.243 | 7.113 |
| 18.5 | -0.70 | -0.53 | -0.34 | -0.18 | -0.01 | 0.15 | 0.32 | 0.51 | 0.68 | 0.234 | 7.518 |
| 19.0 | -0.65 | -0.49 | -0.31 | -0.16 | 0.00 | 0.16 | 0.31 | 0.49 | 0.65 | 0.224 | 7.791 |
| 19.5 | -0.62 | -0.46 | -0.29 | -0.14 | 0.01 | 0.16 | 0.31 | 0.48 | 0.64 | 0.215 | 7.828 |
| 20.0 | -0.58 | -0.43 | -0.27 | -0.12 | 0.02 | 0.17 | 0.31 | 0.48 | 0.63 | 0.207 | 7.563 |

# References

1. Cole T, Green P (1992) Smoothing reference centile curves: The LMS method and penalized likelihood. Statistics in medicine

2. Rigby RA, Stasinopoulos DM (2005) Generalized additive models for location, scale and shape. Journal of the Royal Statistical Society: Series C (Applied Statistics) 54(3):507–554. doi: 10.1111/j.1467-9876.2005.00510.x

3. Royston P, Wright EM (2000) Goodness-of-fit statistics for age-specific reference intervals. Statistics in medicine 19(21). doi: 10.1002/1097-0258(20001115)19:21<2943:aid-sim559>3.0.co;2-5

4. van BS, Fredriks M (2001) Worm plot: A simple diagnostic device for modelling growth reference curves. Statistics in medicine 20(8). doi: 10.1002/sim.746

5. De Onis M (2006) WHO Child Growth Standards - Length/Height-for-age, Weight-for-age, Weight-for-length, Weight-for-height and Body Mass Index-for age: Methods and Development. World Health Organization, Geneva

6. Duran I, Martakis K, Stark C, Ballmann M, Hamacher S, Schoenau E et al. (2019) Suitability of growth standards for growth monitoring in children with genetic diseases. Anthropologischer Anzeiger; Bericht uber die biologisch-anthropologische Literatur 76(1):15–28. doi: 10.1127/anthranz/2019/0932
