# Supplementary material for: Multivariable reference centiles for maximum grip strength in childhood to young adults
Source: Eur J Clin Nutr. 2023 Dec 29;78(6):494–500. doi: 10.1038/s41430-023-01395-4 (PMC11182743; doi:10.1038/s41430-023-01395-4)
Supplement: Supplementary file 2 — Supplement Figure legends [file 41430_2023_1395_MOESM2_ESM.pdf]

Supplement Figure legends

Suppl. Figure 1 Bland-Altman diagram of the prediction accuracy of the multiple linear regression

Bland-Altman diagram of the prediction accuracy of the multiple linear regression to predict mGS in females (A) and males (B) were presented.

Suppl. Figure 2

In order to visualize the presented multivariable reference centiles for females more clearly, averaged "projections" were generated for fixed ranges for height and BMI and compared with the classic, only sex- and age-adjusted, reference centiles (details see Methods).

The range of the centiles for height and BMI was given in the title of each subfigure.

The X-axis represents age, and the Y-axis represents maximum grip strength.

Suppl. Figure 3

In order to visualize the presented multivariable reference centiles for males more clearly, averaged "projections" were generated for fixed ranges for height and BMI and compared with the classic, only sex- and age-adjusted, reference centiles (details see Methods).

The range of the centiles for height and BMI was given in the title of each subfigure.

The X-axis represents age, and the Y-axis represents maximum grip strength.

Suppl. Figure 4

Reference centiles for the change of mGS z-scores for females were presented in A for mGS z-scores which were sex- and age-adjusted and in B which were sex-, age-, height- and BMI-adjusted.

Reference centiles for the change of mGS z-scores for males were presented in C for mGS z-scores which were sex- and age-adjusted and in D which were sex-, age-, height- and BMI-adjusted.

Suppl. Figure 5

The solid line represents the false positive classification rate having a low mGS, using only sex- and age-adjusted z-scores. The dashed line represents the false negative classification rate having a low mGS, using only sex- and age-adjusted z-scores. The X-axis represents the sum of the age-adjusted z-scores for height and BMI.

The false positive classification rate increased with decreasing z-scores for height and BMI.

The false negative classification rate increased with increasing z-scores for height and BMI.

For further details s. Methods and Discussion.

Suppl. Figure 6

In each subfigure the 10th, 50th and the 90th centile were given.

In subfigure A and B our sex- and age- adjusted reference centiles for mGS were compared with those of Dodds et al. In subfigure C and D our reference centiles were compared with those of Ploegmakers et al. In subfigure E and F our reference centiles were compared with those of Kocher et al.
