# Supplementary figures and images for: Multivariable reference centiles for maximum grip strength in childhood to young adults
Source: Eur J Clin Nutr. 2023 Dec 29;78(6):494–500. doi: 10.1038/s41430-023-01395-4 (PMC11182743; doi:10.1038/s41430-023-01395-4)

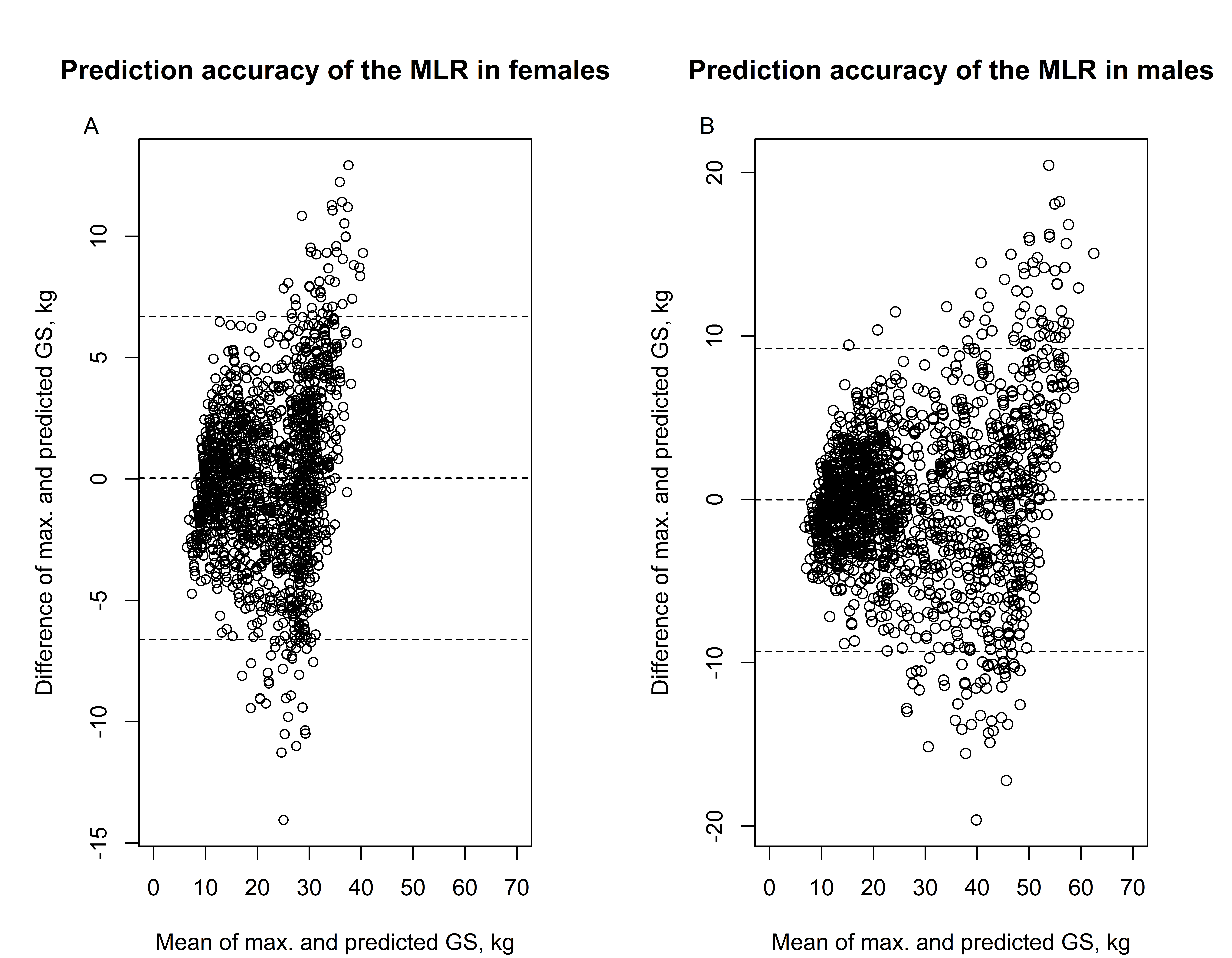

Supplement: Supplementary file 3 — Supplement Fig. 1 [file 41430_2023_1395_MOESM3_ESM.tif]

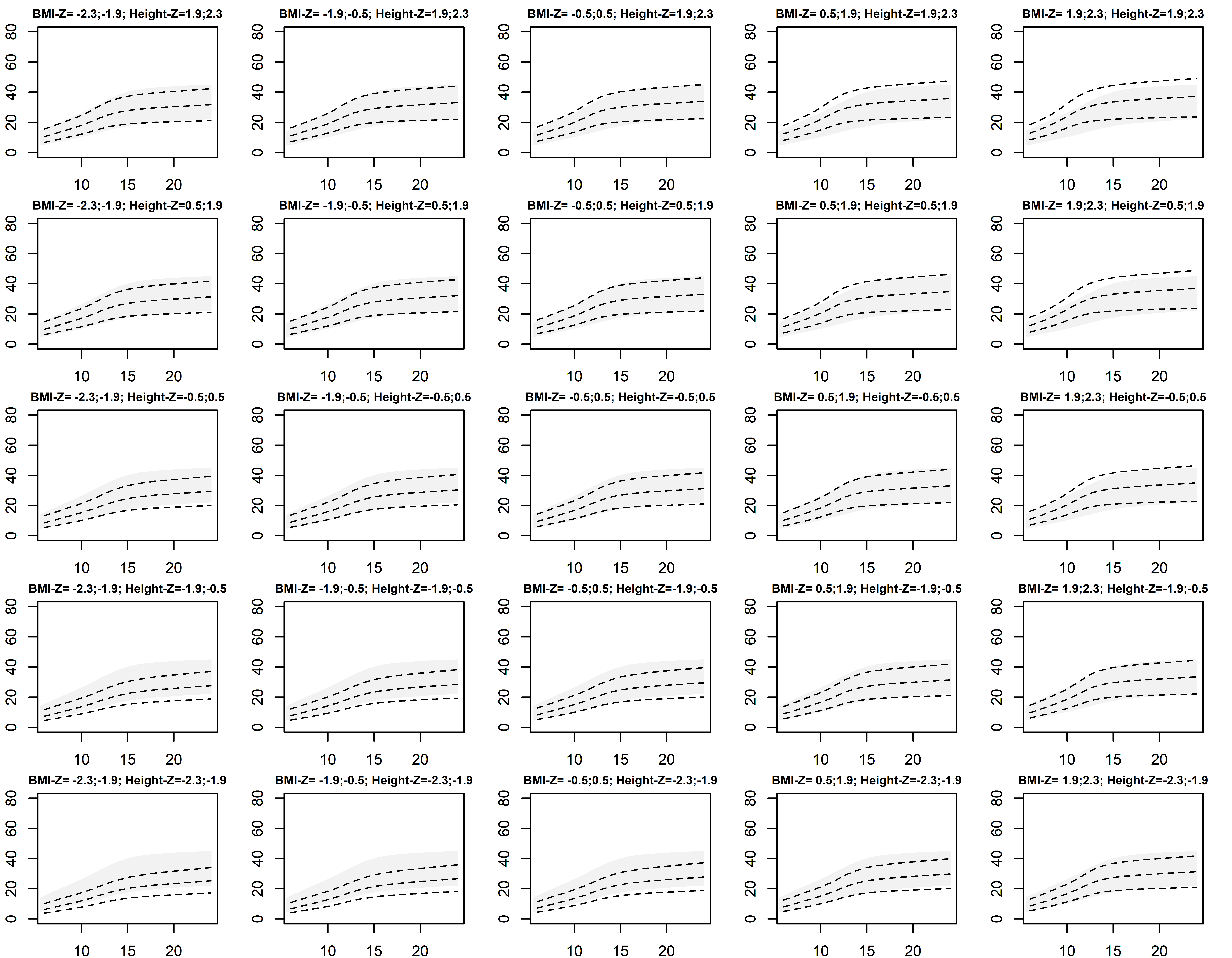

Supplement: Supplementary file 4 — Supple. Fig. 2 [file 41430_2023_1395_MOESM4_ESM.tif]

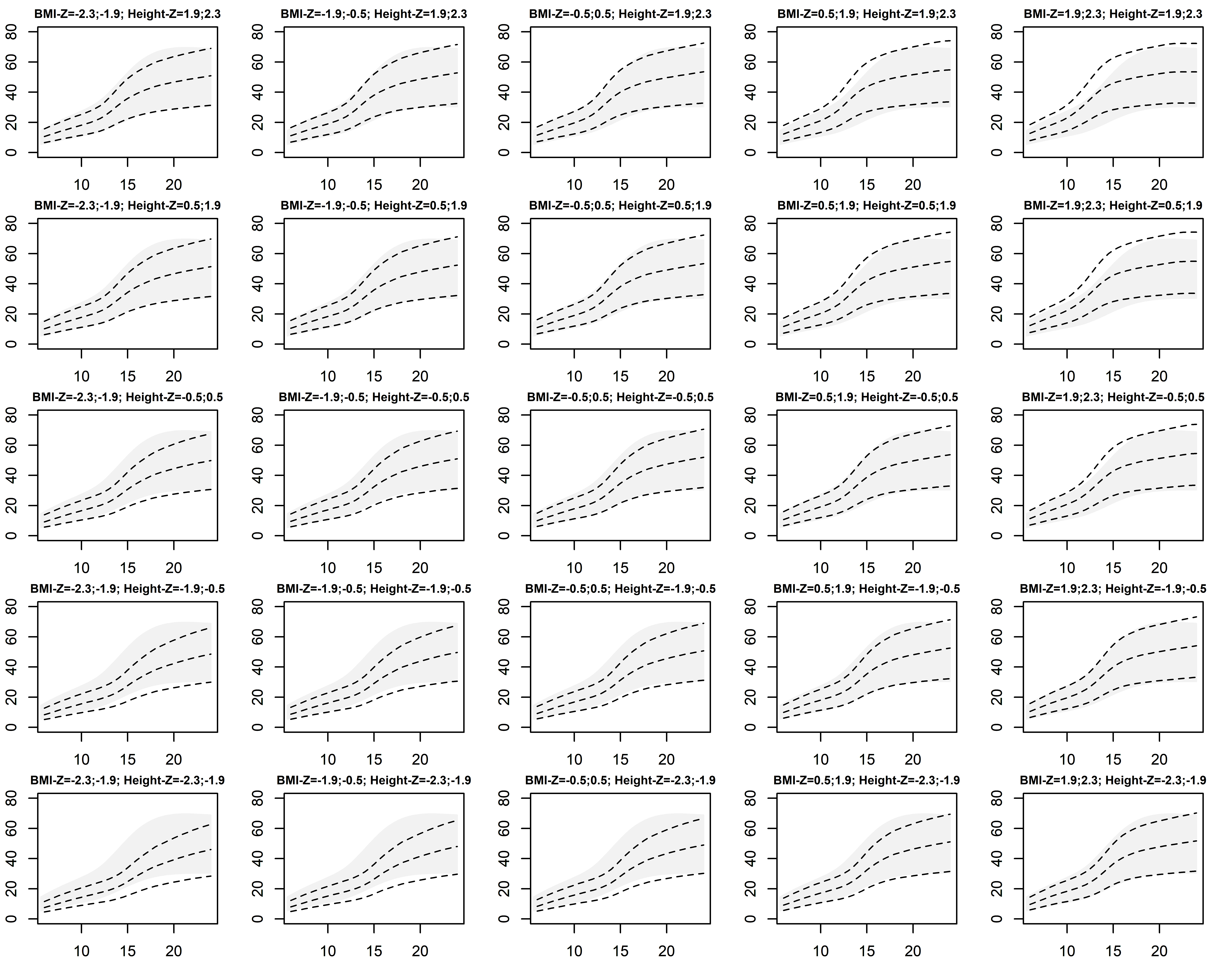

Supplement: Supplementary file 5 — Supple. Fig. 3 [file 41430_2023_1395_MOESM5_ESM.tif]

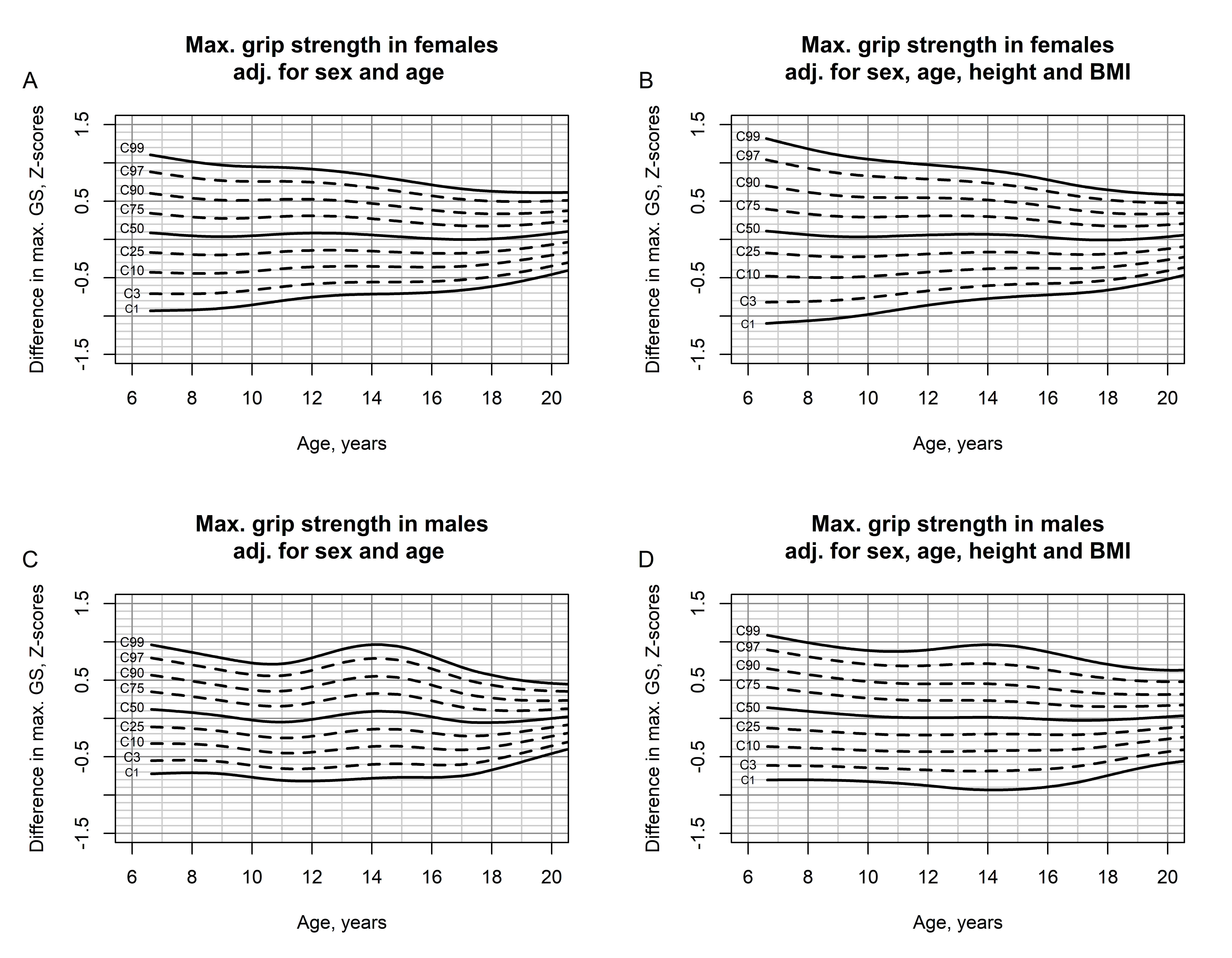

Supplement: Supplementary file 6 — Supple. Fig. 4 [file 41430_2023_1395_MOESM6_ESM.tif]

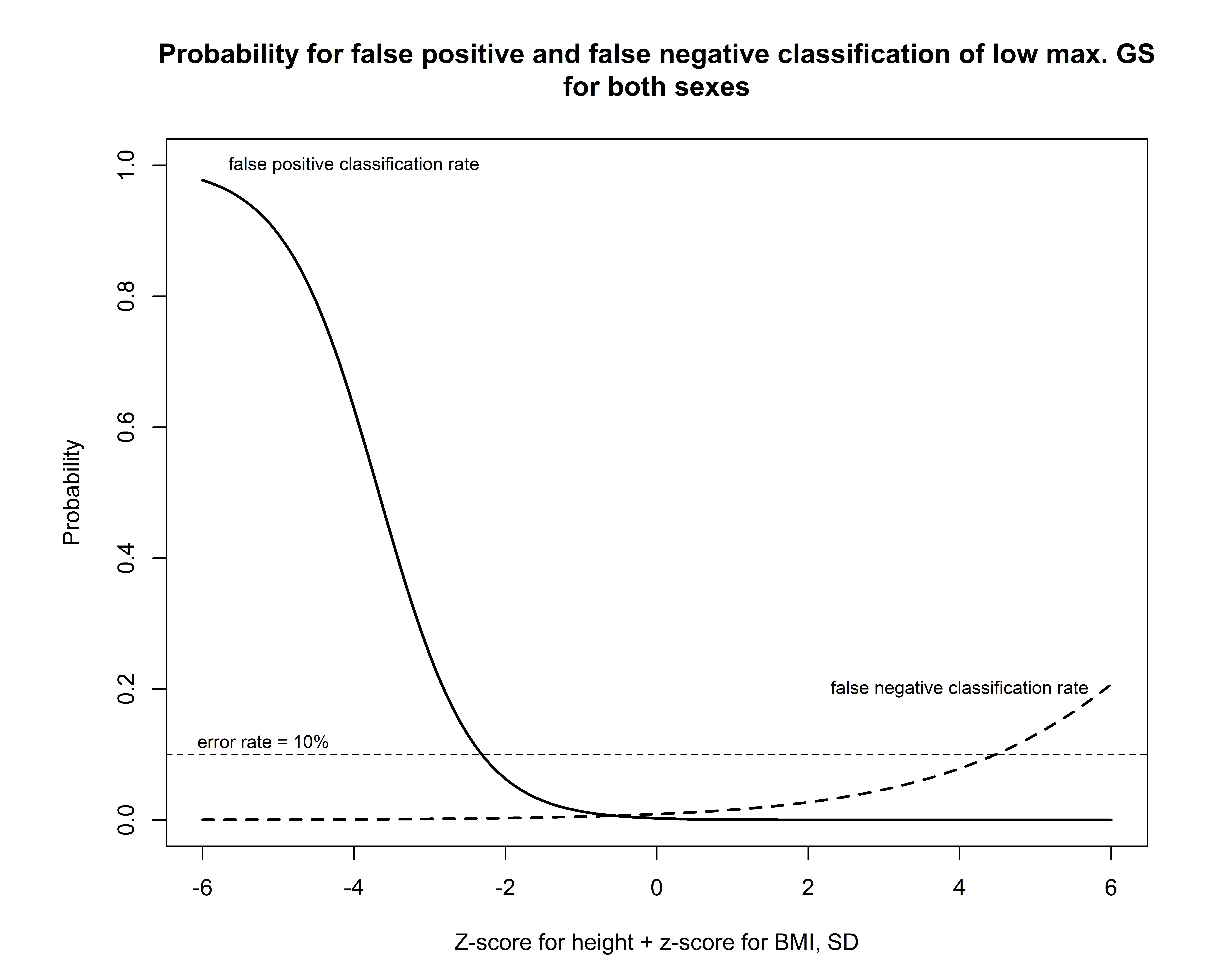

Supplement: Supplementary file 7 — Supple. Fig. 5 [file 41430_2023_1395_MOESM7_ESM.tif]

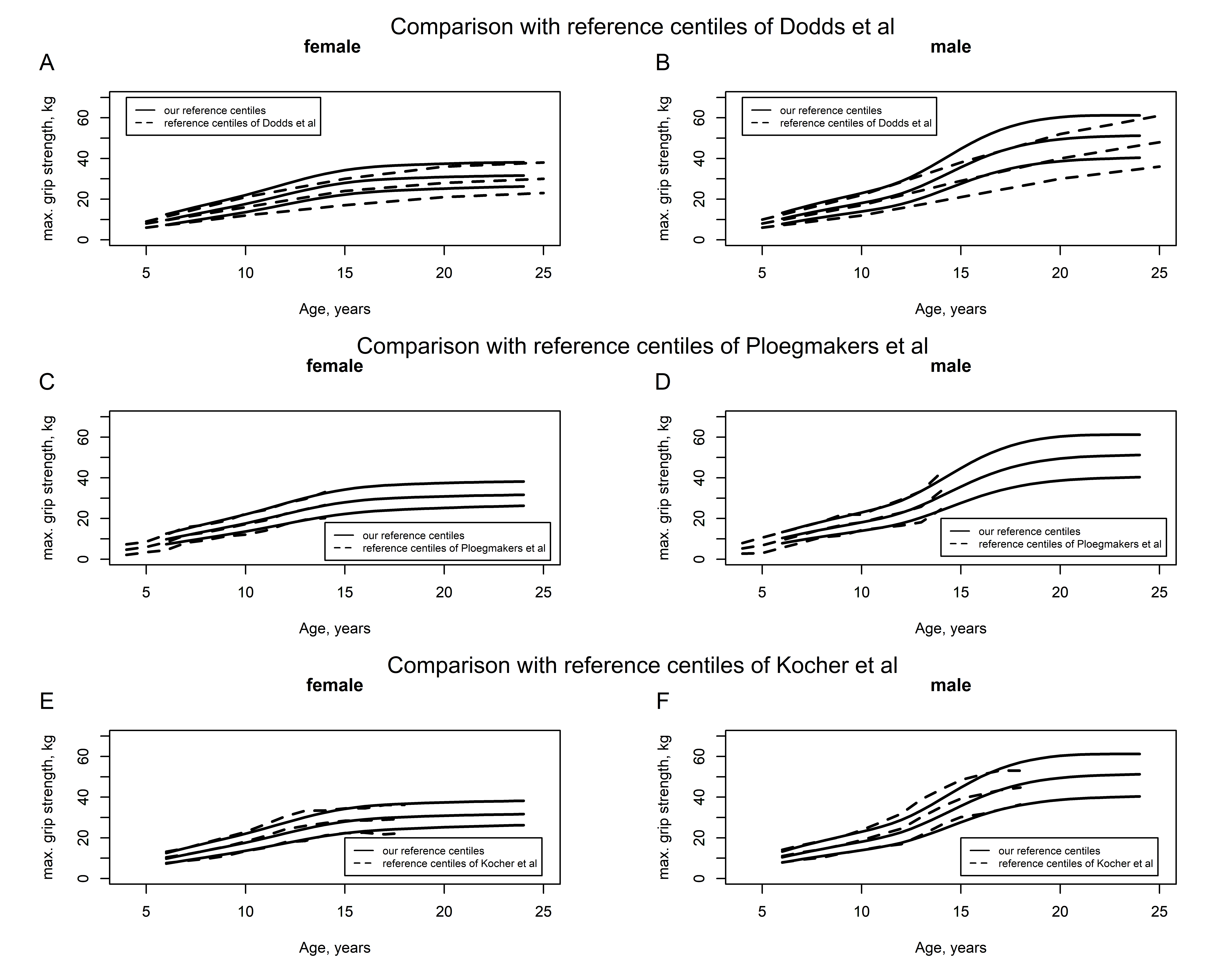

Supplement: Supplementary file 8 — Supple. Fig. 6 [file 41430_2023_1395_MOESM8_ESM.tif]
